# Matefin/SUN-1 Phosphorylation Is Part of a Surveillance Mechanism to Coordinate Chromosome Synapsis and Recombination with Meiotic Progression and Chromosome Movement

**DOI:** 10.1371/journal.pgen.1003335

**Published:** 2013-03-07

**Authors:** Alexander Woglar, Anahita Daryabeigi, Adele Adamo, Cornelia Habacher, Thomas Machacek, Adriana La Volpe, Verena Jantsch

**Affiliations:** 1 Department of Chromosome Biology, Max F. Perutz Laboratories, University of Vienna, Vienna, Austria; 2 CNR Institute of Genetics and Biophysics “A. Buzzati-Traverso,” Napoli, Italy; Imperial College, United Kingdom

## Abstract

Faithful chromosome segregation during meiosis I depends on the establishment of a crossover between homologous chromosomes. This requires induction of DNA double-strand breaks (DSBs), alignment of homologs, homolog association by synapsis, and repair of DSBs via homologous recombination. The success of these events requires coordination between chromosomal events and meiotic progression. The conserved SUN/KASH nuclear envelope bridge establishes transient linkages between chromosome ends and cytoskeletal forces during meiosis. In *Caenorhabditis elegans*, this bridge is essential for bringing homologs together and preventing nonhomologous synapsis. Chromosome movement takes place during synapsis and recombination. Concomitant with the onset of chromosome movement, SUN-1 clusters at chromosome ends associated with the nuclear envelope, and it is phosphorylated in a *chk-2-* and *plk-2*-dependent manner. Identification of all SUN-1 phosphomodifications at its nuclear N terminus allowed us to address their role in prophase I. Failures in recombination and synapsis led to persistent phosphorylations, which are required to elicit a delay in progression. Unfinished meiotic tasks elicited sustained recruitment of PLK-2 to chromosome ends in a SUN-1 phosphorylation–dependent manner that is required for continued chromosome movement and characteristic of a zygotene arrest. Furthermore, SUN-1 phosphorylation supported efficient synapsis. We propose that signals emanating from a failure to successfully finish meiotic tasks are integrated at the nuclear periphery to regulate chromosome end–led movement and meiotic progression. The single unsynapsed X chromosome in male meiosis is precluded from inducing a progression delay, and we found it was devoid of a population of phosphorylated SUN-1. This suggests that SUN-1 phosphorylation is critical to delaying meiosis in response to perturbed synapsis. SUN-1 may be an integral part of a checkpoint system to monitor establishment of the obligate crossover, inducible only in leptotene/zygotene. Unrepaired DSBs and unsynapsed chromosomes maintain this checkpoint, but a crossover intermediate is necessary to shut it down.

## Author Summary

During meiosis, one round of replication is followed by two divisions to produce haploid gametes. During the first meiotic division, faithful segregation is facilitated by the formation between the parental homologs of a physical tether called crossover. Crossovers require the introduction of DNA double-strand breaks, chromosome pairing, formation of the synaptonemal complex, and double-strand break repair by homologous recombination using the homolog as a repair template. This process is accompanied by chromosome movement mediated by the conserved SUN/KASH bridge, which spans the nuclear envelope and connects chromosomes in the nucleus to cytoplasmic forces. These forces stir chromosomes, helping to bring homologs together and to inhibit undesired interactions. Unfaithful chromosome segregation leads to infertility, pregnancy loss, and conditions linked to mental retardation. Chromosome synapsis and DSB repair need to be coordinated with meiotic progression. In this study, we present evidence that Matefin/SUN-1 in *C. elegans* is part of a meiotic surveillance mechanism that monitors events leading to crossing over and thus coordinates meiotic progression and chromosome movement with establishment of the obligate crossover.

## Introduction

Sexually reproducing organisms must halve their genome prior to gamete formation to maintain genome size in succeeding generations. During meiosis, one cycle of DNA replication is followed by two successive rounds of nuclear divisions, resulting in four nuclei that can develop into haploid gametes. During the first meiotic division, homologous chromosomes are segregated from one another; this process depends on the formation of chiasmata, the physical linkages between homologous chromosomes that are generated by crossovers. Crossovers are created by the repair of programmed DNA double-strand breaks (DSBs), with the homologous chromosome used as a repair template. In addition to processing of DSBs, crossover formation requires the completion of earlier events that pair and put into close proximity the homologous chromosomes. In most organisms, the stable juxtaposing of homologs along their length is established by the synaptonemal complex (SC), a proteinaceous structure that forms between them (for review, see [1]).

In the syncytial gonad of the round worm *Caenorhabditis elegans*, meiotic prophase I nuclei are organized in a temporal and spatial manner from meiotic entry to diakinesis, where they pause before entering the spermatheca as cellularized oocytes. Germ cells migrate through the germline syncytium at a rate of one cell row per hour as they pass through the different stages of prophase I [2]. Thus, the *C. elegans* gonad represents a meiotic time course in which landmark events can be investigated.

Pairing and synapsis between homologous chromosomes occurs after meiotic entry in the transition zone (TZ), which corresponds to the leptotene/zygotene stages of meiosis. A class of zinc finger proteins, autosomal ZIM-1, -2, and -3 and X-chromosome-specific HIM-8 (collectively referred to as ZIMs), bind in a *chk-2*-dependent manner to short, repetitive sequences at one subtelomeric region on each chromosome; these are known as homology recognition regions or pairing centers [3]–[10]. ZIMs bound to their corresponding pairing center are seen in the TZ as distinct foci at the nuclear envelope. These foci recruit the polo kinase PLK-2 [11], [12], which is responsible for the movement of chromosomes in a restricted region of the nuclear envelope throughout the TZ, resulting in a crescent-shaped chromatin appearance [11]–[14]. Movement of meiotic chromosomes mediates the pairing center/ZIM-dependent pairing of homologous chromosomes and their homologous synapsis, and it prevents synapsis between nonhomologous chromosomes [11]–[13], [15]. Studies from other organisms support the model that meiotic chromosome movement is required to ensure the removal of undesired chromosome entanglements during recombination and formation of the synaptonemal complex (for review, see [16]).

Kinetic forces generated in the cytoplasm are responsible for chromosome movement and bridging of the nuclear envelope; transmission of these cytoplasmic forces to chromosomes to generate movement contributes to the formation of the evolutionarily conserved meiotic chromosome bouquet. The bouquet is defined as a configuration in which telomeres anchored within the nuclear envelope locally cluster during meiotic chromosome pairing stages (for review, see [17]). In *C. elegans*, leptotene/zygotene chromosomes are tethered to the nuclear envelope at their pairing centers, where tubulin/dynein-dependent chromosome movements are mediated by a SUN/KASH nuclear envelope bridge to the cytoplasm [13], [15], [18].

Matefin/SUN-1 is the inner nuclear envelope transmembrane constituent of this bridge. The N-terminal nucleoplasmic domain interfaces with chromatin, and the perinuclear C terminus interacts with the KASH (Klarsicht/ANC-1/Syne homology) domain of ZYG-12, the outer nuclear envelope component of this bridge (for review, see [19], [20]). SUN-1 and ZYG-12 are evenly distributed throughout the nuclear envelope in mitotic germline cells [13], [14]. At the onset of leptotene/zygotene, PLK-2 recruitment to the pairing centers at the nuclear envelope induces highly mobile aggregation of SUN-1 and ZYG-12 at the pairing center–bearing chromosome ends [11]–[15]. This coincides with *chk-2-* and *plk-1/2*-dependent phosphorylation of SUN-1 at several residues located within its nuclear N-terminal portion [11], [12], [14]. This phosphorylation is independent of chromosome movement, pairing, and establishment of the SUN/KASH bridge [11]. In *chk-2* mutants, chromosome mobilization is absent; this can be explained by the failure to recruit PLK-2 to pairing centers [11], [12], [14].

After several hours of localized movements and SC elongation along chromosome axes, the nuclei enter early pachytene, where the chromosomes are found in a more dispersed configuration [21]. The SUN-1 aggregates, colocalized with autosomal pairing centers, are dissolved in early pachytene. The last remaining aggregate that colocalizes with a HIM-8 focus dissolves in mid-pachytene, accompanied by the complete dephosphorylation of SUN-1 and complete dispersal of chromatin throughout the nuclear volume [14].

As the chromosomes pair and synapse, the early steps of crossover formation are also initiated. In many organisms, the search for a homologous repair template is aided by “single-strand DNA feelers” derived from processed DSBs to drive meiotic pairing through a localized search for homology. In the worm, DSBs are thought to be introduced in leptotene/zygotene [22] and are repaired over the course of pachytene to give rise to the obligate crossover, a process that depends on the SC [23], [24].

Prior to anaphase I, multiple checkpoint mechanisms ensure faithful transmission of chromosomes (for review, see [25]). In the *C. elegans* germline, unrepaired DNA lesions lead to *cep-1*/p53-dependent apoptosis or a meiotic progression delay [26]–[28]. Unsynapsed chromosomes can trigger damage-independent apoptosis, which depends on *pch-2*, ZIMs, and *plk-2* [12], [29]. Furthermore, synaptic failure triggers an arrest in the leptotene/zygotene stage, which leads to the persistence of SUN-1 aggregates, chromosome movement, and a clustered chromatin conformation [14], [21], [30].

In this study, we addressed the role of SUN-1 phosphorylation during prophase I of *C. elegans* meiosis. We found that failure to successfully execute meiotic tasks (such as synapsis or recombination) leads to persistent SUN-1 phosphorylation, and that this phosphorylation is required to initiate a meiotic progression delay under these challenged conditions. We demonstrate that in the male gonad (X0), this mechanism is switched off for the single, and therefore unsynapsed, X chromosome. Furthermore, we demonstrate that SUN-1 phosphomodifications are required for SUN-1 aggregate stability, continued PLK-2 localization to pairing centers, and formation of the SC with wild-type kinetics. These data lead us to propose that phosphorylation of the SUN-1 nucleoplasmic domain integrates signals emanating from processes pivotal to the formation of the obligate crossover with meiotic progression and chromosome movement.

## Results

### SUN-1 phosphomodifications

**Figure 1:**
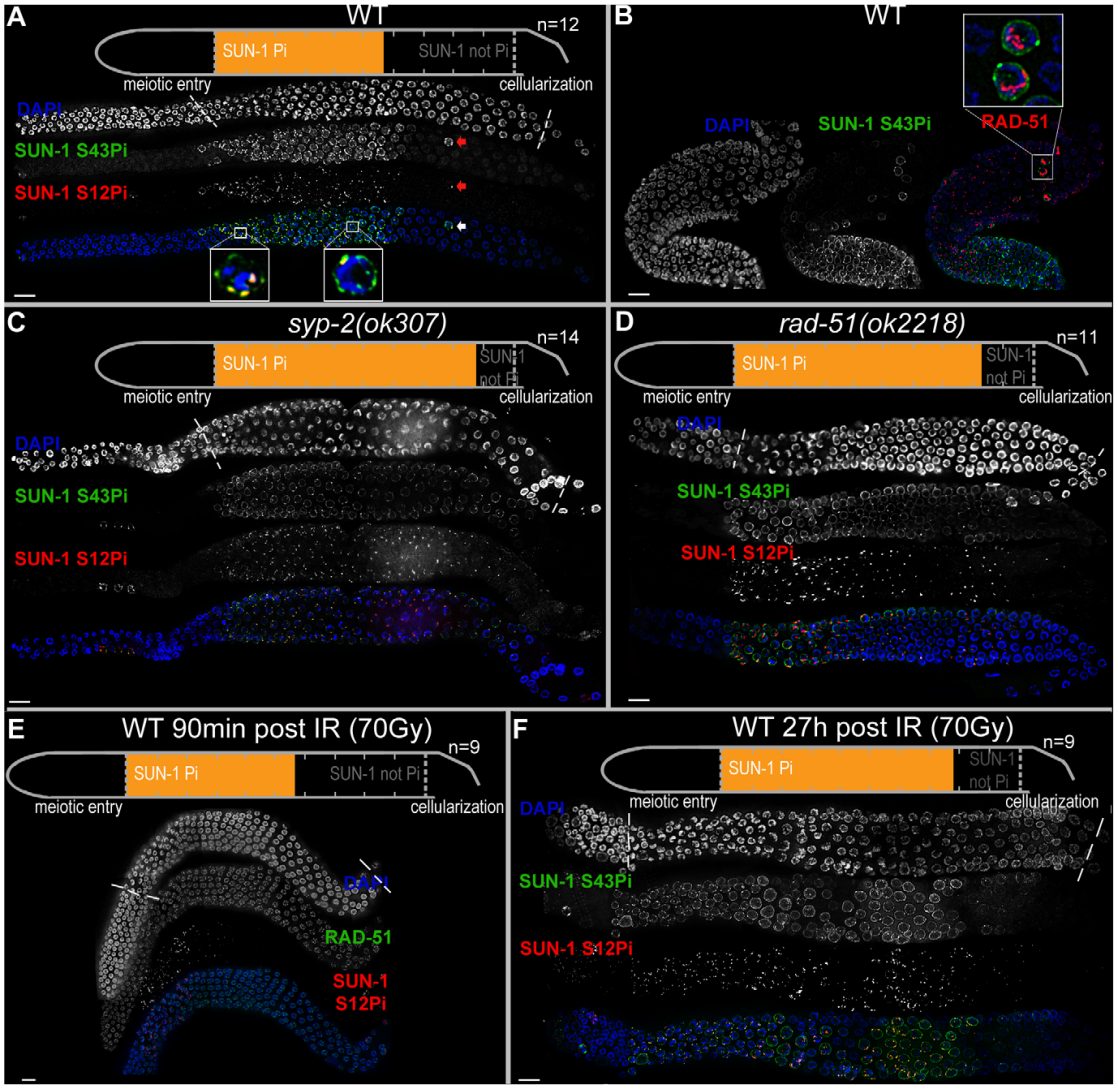
Prolonged SUN-1 phosphorylation correlates with meiotic failure. (A) Wild-type (WT) hermaphrodite gonad stained with DAPI (top and blue in merge), anti-SUN-1 S43Pi (middle and green in merge), and anti-SUN-1 S12Pi (bottom and red in merge). Arrow highlights a nucleus in mid/late pachytene zone with clustered chromatin and phosphorylated SUN-1. Magnifications at the bottom of representative TZ or early pachytene nuclei to highlight differences in SUN-1 phosphorylation patterns. Schematics on top (in A, C, D, and E) delineate quantifications of nuclei with and without phosphorylation of SUN-1 (S8, S12, S24, and S43) in the meiotic part of the gonad (quantified from meiotic prophase entry to beginning of cellularization/diplotene in WT, marked with dotted lines in DAPI channels). Orange represents cell rows with ≥50% of nuclei with SUN-1 phosphorylation. *n*, number of gonads scored for each genotype. (B) Wild-type hermaphrodite gonad stained with DAPI (left and blue in merge), anti-SUN-1 S43Pi (middle and green in merge), and anti-RAD-51 (red in merge). Box, upper right: magnification of representative nuclei in late pachytene with clustered chromatin, phosphorylated SUN-1, and numerous RAD-51signals. Red channel boosted in merge picture to better visualize RAD-51 foci in all nuclei. (C and D) *syp-2(ok307)* (C) and *rad-51(ok2218)* (D) mutant hermaphrodite gonad stained with DAPI (top and blue in merge), anti-SUN-1 S43Pi (middle and green in merge), and anti-SUN-1 S12Pi (bottom and red in merge). (E) Wild-type hermaphrodite gonad dissected 90 min after 90 Gy gamma irradiation stained with DAPI (top and blue in merge), anti-RAD-51 (middle and green in merge), and anti-SUN-1 S12Pi (bottom and red in merge). (F) Wild-type hermaphrodite gonad dissected 27 h after 70 Gy gamma irradiation; anti-SUN-1 S12Pi (red), anti-SUN-1 S43Pi (green), and DAPI (blue). Scale bars, 10 µm.

SUN-1 is phosphorylated on seven nucleoplasmic, N-terminal residues: S8, S12, S24, S43, S58, S62, and S35 or T36 [14]. Four cytologically active antibodies against phosphorylated S8, S12, S24, and S43 [11], [14] were generated and used in previous studies. Here, we briefly summarize their properties in wild-type worms (Figure 1A and Figure S1). Meiotic SUN-1 phosphorylations on S8, S12, S24, and S43 appear synchronously with entry into leptotene/zygotene [11], [14]. S8, S24, and S43 are phosphorylated on the entire population of SUN-1, whereas S12 is exclusively phosphorylated on the population of SUN-1 found in aggregates at the nuclear envelope–attached chromosome ends. All phosphorylations are dependent on *chk-2*; however, they are independent of *spo-11* and *atm-1/atr-1* [14]. *plk-2* is exclusively required to phosphorylate SUN-1 on S12, and *plk-1* can partly substitute for *plk-2*. Additionally, SUN-1 aggregate formation depends on *plk-1/2*, and homologous pairing is strongly affected in *plk-2* mutants [11], [12]. The ZIMs (pairing center proteins of the autosomes) and HIM-8 (X-chromosome-specific pairing center protein) always colocalize with a SUN-1 aggregate [13], [14].

In early pachytene, when all chromosome axes are synapsed, SUN-1 stays phosphorylated on residues 8, 24, and 43. These modifications are found on the entire population of SUN-1; they can be considered equal (for overlap of localization, see Figure S1C and D), and the choice of antibodies to detect them was dictated by the compatibility with other antibodies. SUN-1 phosphorylated on S12 is now limited to the last persisting SUN-1 aggregate that colocalizes with HIM-8. PLK-2 stays localized to this aggregate and starts to relocate to stretches on synapsed chromosomes. This is accompanied by release of the tight chromatin clustering. In mid/late pachytene, both SUN-1 aggregates and SUN-1 phopsphorylation are no longer present. PLK-2 stretches are fully elongated along synapsed chromosomes.

In summary, when, in a given nucleus, SUN-1 is phosphorylated on S8, S24, and S43, the nucleus always displays one or more SUN-1 aggregates. These aggregates are phosphorylated on S12. This is the case for the wild type and for all of the mutants presented in this study. Therefore, when we use the term “SUN-1 phosphorylation” we refer to all phosphorylation events.

### Persistent SUN-1 phosphorylation correlates with synaptic and recombinational errors in the wild type

We observed the occasional appearance of single nuclei that showed persistent phosphorylation of SUN-1 in the mid/late pachytene zone in the gonad (Figure 1A, arrow). On average, we counted 2.6 (standard deviation [SD] 2.2) such nuclei in the mid/late pachytene region of wild-type gonads (*n* = 46). In contrast to the pachytene nuclei surrounding them, these nuclei had a tightly clustered, leptotene/zygotene-like chromatin configuration, were positive for SUN-1 phosphorylation, and had one or more SUN-1 aggregates (positive for S12 phosphorylation). Furthermore, these nuclei also showed high numbers of RAD-51 foci (Figure 1B), consistent with the presence of unrepaired DSBs and occasional synaptic failures (Figure S2A, arrow). This cytological appearance could be triggered by apoptosis, which culls half the nuclei in the late pachytene/diplotene zone of the gonad [31]. Nevertheless, these nuclei were also present in *ced-3(n717)* apoptosis-defective mutants (Figure S2B, arrow), indicating that they do not represent nuclei with an activated apoptotic machinery.

These occasional SUN-1 phosphorylation-positive nuclei seemed to be nuclei that were arrested in their cell cycle progression due to synaptic or recombination failures. This was described previously only for synapsis mutants in *C. elegans* [21]. To gain further support for this hypothesis, we tested for the presence of phosphorylated CHK-1, an indicator of checkpoint activation as described by Jaramillo-Lambert et al. [27]. The stray leptotene/zygotene-characteristic nuclei in mid/late pachytene displayed the highest levels of CHK-1 phosphorylation in the gonad (Figure S2C). We therefore hypothesize that SUN-1 phosphorylation, aggregate persistence, and associated chromosome mobility is sustained in inappropriate late stages in response to the presence of unfinished meiotic tasks.

Phosphorylated CHK-1 signals overlap strikingly with phosphorylated SUN-1 from meiotic entry onwards (Figure S2C). The signal intensity we observed is stronger than was described previously [27]. CHK-1 is phosphorylated and thereby activated by the damage signal kinases ATM and ATR (*atl-1* in worms) [32]. Consistently, *atm-1; atl-1* double mutants were devoid of phosphorylated CHK-1 signals (Figure S2D). Because SUN-1 phosphorylation still occurs in *atm-1*; *atl-1* [14], we reasoned that signals from meiotic damage must be transmitted through multiple parallel pathways to CHK-1, CHK-2, and SUN-1.

### Synaptic and recombination failure independently prolong phosphorylation of SUN-1

To understand the nature of the failure leading to prolonged SUN-1 phosphorylation in the stray leptotene/zygotene-like nuclei late in the gonad, we compared SUN-1 phosphorylation, SUN-1 aggregate persistence, and chromatin morphology between wild-type and mutant worms that were blocked in different steps of meiotic recombination.

We defined and measured the meiotic region of the germline between meiotic entry (marked by chromatin clustering) and diplotene (marked by cellularization of the oocytes; dotted lines in Figure 1A and1C–F). In the wild type, the distal 56.3±3.8% of the meiotic region was populated by TZ or early pachytene nuclei displaying fully phosphorylated SUN-1 (S8, S12, S24, and S43), one or more SUN-1 aggregates, and clustered chromatin. The proximal 43.8±3.8% of the gonad showed mainly mid/late pachytene nuclei without SUN-1 phosphorylation or aggregates (Figure 1A and Table 1).

**Table 1:** Length of SUN-1 phosphorylation in wild-type and mutant backgrounds.

Genotype	Percentage of cell rows with SUN-1 phosphorylation (normalized to gonad length)	*p* values (wild type as reference)	*n*
WT	56.25±3.8	—	12
*syp-2(ok307)*	85±4.8	9.45E-15	14
*rad-51(ok2218)*	82.2±6.1	3.55E-11	11
*spo-11(ok79)*	77.5±5.6	4.75E-10	11
*spo-11(ok79); syp-2(ok307)*	87.3±4.7	4.75E-14	11
WT 2 h post-70 Gy	57.7±4.1	0.4	9
WT 8 h post-70 Gy	65.6±5.3	1.46E-5	9
WT 24–27 h post-70 Gy	78.3±6.6	8.54E-09	9

Dissected gonads were measured from meiotic entry (TZ) to beginning of cellularization. Relative percentage of cell rows with SUN-1 phosphorylation was assessed and normalized to the length of the meiotic gonad from meiotic entry to cellularization. When >50% of nuclei in a cell row were phosphorylated on SUN-1, it was counted as phosphorylated. Variations correspond to the standard deviation. *p* values indicate comparison of percentage of cell rows with SUN-1 phosphorylation between wild type and the respective mutant in a two-tailed *t*-test. *n*, number of hermaphrodites scored; WT, wild type.

We first examined the effect of loss of synapsis on SUN-1 phosphorylation. *syp-2* mutants are unable to form an SC due to loss of the central element component. In *syp-2* mutants, repair of DSBs is strongly delayed and never gives rise to a crossover. Leptotene/zygotene-like clustered chromatin persists [24]. Both SUN-1 aggregates (surrounding autosomes and the X chromosome) and phosphorylation persisted until late in the gonad in *syp-2(ok307)* mutants and comprised 85±4.8% of the “meiotic gonad” (Figure 1C, Table 1, and [14]). These features of a progression delay were not abrogated in *syp-2; spo-11* double mutants, which do not form meiotic DSBs (Table 1). This result shows that synapsis defects are sufficient to prolong early prophase SUN-1 phosphorylation and other leptotene/zygotene-like characteristics.

To investigate whether unrepaired DSBs, independent of synaptic failures, can also trigger prolonged SUN-1 phosphorylation, we examined *rad-51(ok2281)* mutants. In this background, the SC assembles normally, but unrepaired DSBs persist [23], [33]. In *rad-51(ok2218)* germlines, the SUN-1 aggregates that colocalized with autosomal pairing centers disappeared with wild-type kinetics upon exit from the TZ. In contrast, the zone of nuclei showing one SUN-1 aggregate colocalizing with the X-chromosomal pairing center was prolonged over almost the entire meiotic gonad. Furthermore, early pachytene-like chromatin clustering persisted in *rad-51(ok2218)* germlines (Figure 1D and Table 1).

### SUN-1 cannot be rephosphorylated in pachytene, and a recombination intermediate is required for dephosphorylation

Since the presence of unrepaired DSBs in *rad-51* and *syp-2* mutants resulted in persistent SUN-1 phosphomodifications, we tested whether the zone in the gonad that displayed SUN-1 phosphorylation and aggregate(s) could be prolonged by introduction of excess exogenous DSBs. In wild-type gonads subjected to a high dosage of ionizing radiation (70 Gy), the zone of nuclei with SUN-1 phosphorylation was not prolonged 20–180 minutes post-irradiation (Figure 1E and Table 1). When gonads were dissected 8 h post-irradiation, the zone of nuclei with phosphorylated SUN-1 was prolonged to 65.6±5.3% (compare to the nonirradated reference, 56.3±3.8%; Table 1). Finally, gonads dissected 27 h post-irradiation revealed nuclei with clustered chromatin and phosphorylated and aggregated SUN-1 throughout 78.3±6.6% of the meiotic gonad (Figure 1F and Table 1). Also at 27 h post-irradiation, there was a strong correlation between SUN-1 phosphorylation and abundant RAD-51 signals late in the gonad; the few nuclei without phosphorylation signals also lacked detectable RAD-51 (Figure S2E, arrowheads). Cells migrate with an approximate speed of one cell row per hour [2]; introduction of DNA lesions in TZ/early pachytene nuclei appears to have led to their inability to exit the stage of SUN-1 phosphorylation and chromatin clusters, despite their migration through the gonad. In contrast, when DSBs were introduced in mid/late pachytene nuclei, SUN-1 was not rephosphorylated and TZ/early pachytene characteristics could not be reestablished.

**Figure 2:**
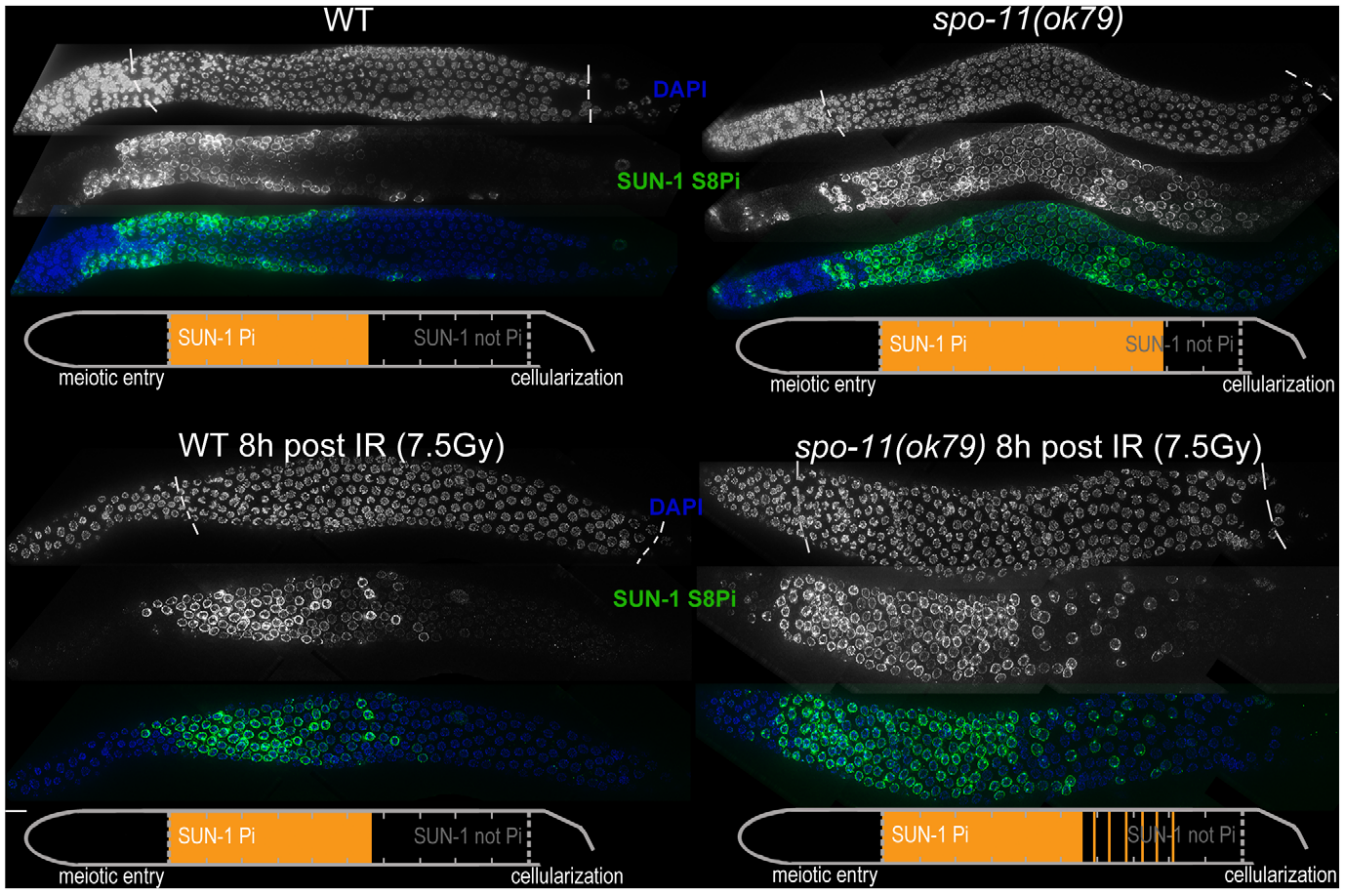
SUN-1 phosphorylation is prolonged in the absence of DSBs and can be decreased to wild-type length by low-dosage irradiation. WT (left) and *spo-11(ok79)* (right) hermaphrodite gonad, not irradiated (top) or irradiated with 7.5 Gy (bottom) dissected 8 h post-irradiation and stained with DAPI (top, blue in merge) and anti-SUN-1 S8Pi (bottom, green in merge). Schematics on the bottom of each gonad represent nuclei with and without phosphorylation of SUN-1 (S8, S12, S24, and S43) in the meiotic part of the gonad (from meiotic prophase entry to beginning of cellularization/diplotene in WT, marked with dotted lines in DAPI channels). Orange represents cell rows with ≥50% of nuclei with SUN-1 phosphorylation.

Pairing and formation of the SC between homologous chromosomes occurs independent of SPO-11-generated meiotic DSBs in *C. elegans* [34]. Surprisingly, we observed that *spo-11(ok79)* mutants with fully elongated SCs and no DSBs showed an extended zone with phosphorylated SUN-1 compared to wild-type germlines (Figure 2 and Table 1). In addition, phosphorylated CHK-1 was detectable longer in *spo-11(ok79)* than in wild-type gonads, and the phosphorylated CHK-1 signal overlapped with phosphorylated SUN-1 (Figure S2C). Overall, however, the phosphorylated CHK-1 signals appeared weaker in *spo-11(ok79)* than in wild-type germlines. By subjecting *spo-11(ok79*) mutants to low doses of ionizing radiation (7.5 Gy), the zone with phosphorylated nuclei could be reduced to wild-type length; only one-third of the nuclei were phosphorylated and two-thirds were nonphosphorylated from mid-pachytene onwards (Figure 2). In contrast, in unirradiated *spo-11* control animals, SUN-1 was phosphorylated in almost all nuclei until cellularization (Figure 2). We chose this irradiation dosage because it was too low to generate a massive damage response. Nevertheless, the dosage was high enough to generate sufficient DSBs as substrates for crossover recombination, since 24 h after 7.5 Gy of ionizing radiation, bivalent formation was fully restored in *spo-11(ok79)* mutants to wild-type levels (DAPI signals in diakinesis: wild type/N2: 5.9, *n* = 18; *spo-11(ok79):* 11.6, *n* = 17; and *spo-11(ok79)* irradiated with 7.5 Gy: 6.0, *n* = 21).

Remarkably, even in the absence of DSBs, SUN-1 phosphorylation paralleled by CHK-1 activation was not only initiated but also prolonged. The persistence of nuclei in an early pachytene-like stage could be abrogated by artificially generating a substrate for crossover recombination. Therefore, a yet-unidentified crossover intermediate is required to terminate the signaling that leads to prolongation of early pachytene characteristics, including SUN-1 phosphorylation.

Our results collectively suggest that prolonged phosphorylation of SUN-1, the presence of SUN-1 aggregates, and chromosome mobility (leading to chromatin clustering) correlate with a broad range of unfinished meiotic tasks. First, full synapsis and repair of DSBs were required for SUN-1 dephosphorylation in mid-pachytene. Second, the establishment of a crossover or crossover intermediate was necessary to exit from the early pachytene stage in which phosphorylated SUN-1 was observed. Therefore, we propose that SUN-1 phosphorylation is a component of a meiotic surveillance system that monitors the progression or completion of these meiotic tasks.

### Analysis of SUN-1 phosphosite mutants in meiosis

Our results suggest that sustained SUN-1 phosphorylation correlates with a broad range of unfinished meiotic tasks. To learn more about the role of SUN-1 phosphorylation in the fulfillment of these meiotic tasks, we generated different GFP-tagged *sun-1* phosphosite mutants. These mutants were obtained by using the MosSCI single-copy insertion system [35], and all analyses were performed in the *sun-1(ok1282)* deletion background (see Materials and Methods and Table 2 for a detailed description of the lines). Mutation of specific residues to alanine generated nonphosphorylatable mutants. Mutations to glutamic acid attempted to mimic constitutively phosphorylated forms of SUN-1. When tested for embryonic viability and occurrence of males due to X-chromosomal nondisjunction, all *sun-1* phosphosite substitution lines exhibited no or only very subtle defects under standard laboratory conditions (Table 2).

**Table 2:** Brood size, hatch rate, and X chromosome nondisjunction of *sun-1* phosphosite mutants.

	Brood size	Hatch rate (%)	Males (%)	*n*
*sun-1(wt)*	211.2±24.9	99.2±0.8	0.04±0.12	15
*sun-1(S12E)*	187.6±35.4	94.1±9.8	0.23±0.24	15
*sun-1(6E)*	134.3±54.0*	91.5±4.1*	0.33±0.49	15
*sun-1(S12A)*	245.2±34.9*	98.0±1.6	0.12±0.16	15
*sun-1(allA)*	207.4±42.7	97.4±2.5	0.25±0.16	15

* *p*<0.001 between wild type and the respective mutant in two-tailed *t*-test.

Variations correspond to the standard deviation. Data were assessed over the complete self-fertile period of hermaphrodites at 20°C. *n*, number of hermaphrodites scored.

**Figure 3:**
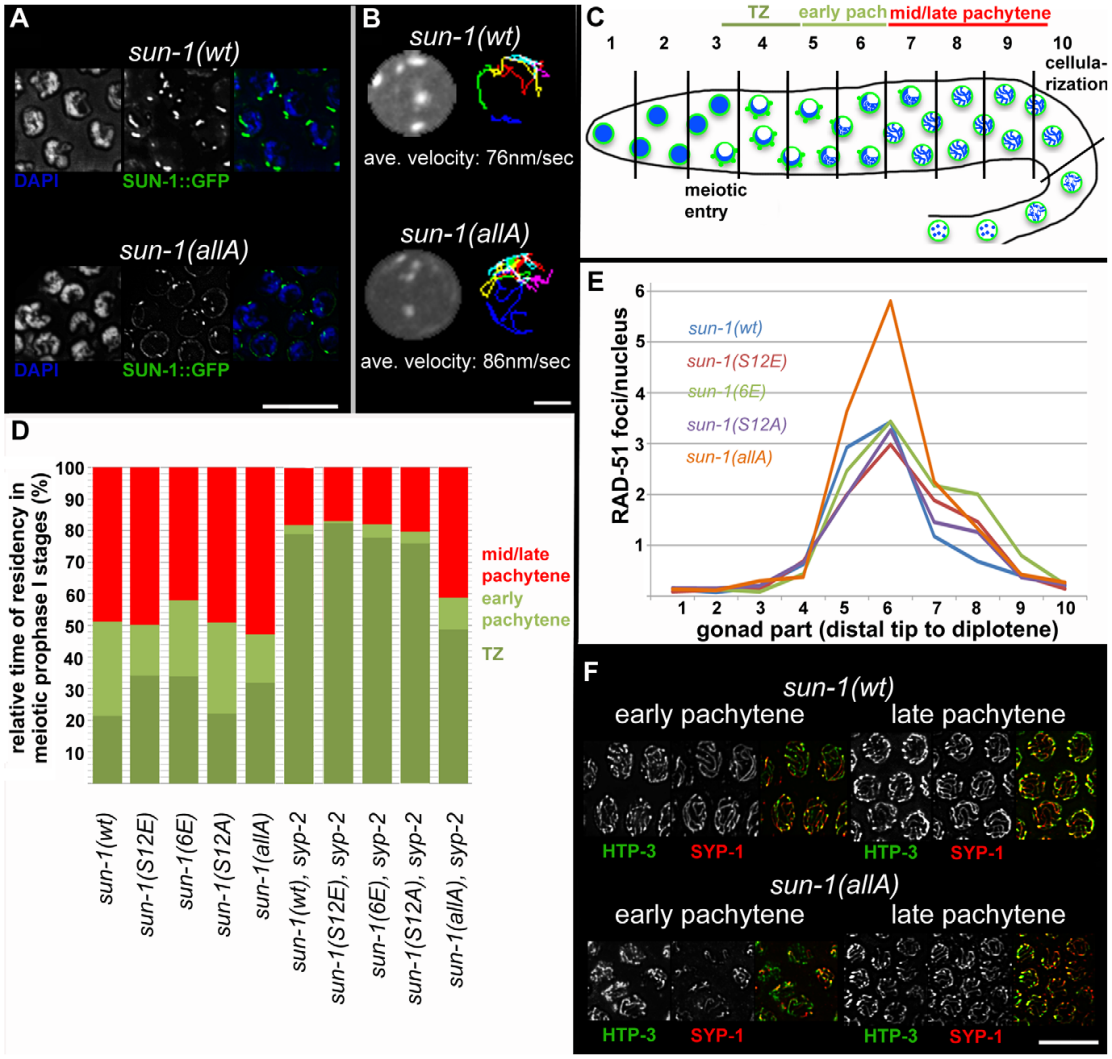
Effect of *sun-1* phosphosite mutations on the duration of meiotic stages, DSB turnover, SUN-1 aggregates, chromosome movement, and synapsis. (A) TZ nuclei of *sun-1(wt); sun-1(ok1282*) (top), and *sun-1(allA); sun-1(ok1282)* (bottom) hermaphrodite gonads stained with DAPI (left; blue in merged picture) and anti-GFP (middle; green in merged picture). Scale bars, 10 µm. (B) First frame of *in vivo* time-lapse GFP-recorded TZ nuclei of *sun-1(wt); sun-1(ok1282)* (top, left) and *sun-1(allA); sun-1(ok1282)* (bottom, left) hermaphrodite gonads. Displacement tracks of SUN-1 aggregates represent 2D plotted chromosome end movements over 3 min (right). Average speed of SUN-1 aggregates in TZ (*n* = 9 nuclei, followed over 3 mins). Scale bar, 2 µm. (C) Schematic of a wild-type hermaphrodite gonad with nuclei in the corresponding zones, as used in the quantifications in Figure 3D: chromatin (blue) and SUN-1 morphology (green). Gonad is subdivided into ten zones, as used for the RAD-51 foci quantification in Figure 3E. Meiotic entry and beginning of cellularization are indicated. (D) Representation of relative time of residency in different meiotic stages, as assessed by presence of SUN-1 aggregates in different mutant backgrounds, normalized to the length of the gonad (from meiotic entry [0%] to start of meiocyte cellularization at diplotene [100%]). Nuclei were sorted into three categories: “TZ” (dark green, more than one SUN-1 aggregate), “early pachytene” (light green, one SUN-1 aggregate), and “late pachytene” (red, no SUN-1 aggregate). Categories were assigned once ≥50% of nuclei in a cell row fulfilled one of these criteria. At least eight gonads were counted per genotype. (E) Average numbers of RAD-51 foci per nucleus in the hermaphrodite gonad of different *sun-1* phosphosite mutants. Gonads were divided into ten zones, as schematically indicated in (B). Three gonads per genotype were counted. (F) Nuclei from early and late pachytene zones of *sun-1(wt); sun-1(ok1282)* (top) and *sun-1(allA); sun-1(ok1282)* (bottom) hermaphrodite gonads stained with anti-HTP-3 (left, green in merged) and anti-SPY-1 (middle, red in merged). Scale bars, 10 µm.

We next asked whether SUN-1 phosphorylation is required to induce movement-competent aggregates at meiotic entry. All *sun-1* phosphosite substitution lines displayed chromatin clustering and were able to form SUN-1 aggregates (unpublished data). Moreover, in the completely nonphosphorylatable mutant *sun-1(allA)*, SUN-1 aggregate formation was not abrogated. Nevertheless, SUN-1 aggregates in a *sun-1(allA)* background were visibly smaller, and less SUN-1 protein was organized in movement-competent aggregates compared to the wild type (Figure 3A). However, *in vivo* time-lapse imaging of *sun-1(allA)* aggregates showed that they remained mobile (Videos S1 and S2) and had wild-type movement characteristics (similar velocities in the TZ, movement along similar distances, and the ability to fuse, coalesce, and separate; elevated numbers of fusion/split events in *sun-1(allA)* were not significant) (Table S1 and Figure 3B). These aggregates also colocalized with ZIMs, as seen in the wild type (Figure S3B).

To address the question of whether SUN-1 phosphorylation influences cell cycle progression, we measured the duration of leptotene/zygotene (TZ), early pachytene, and mid/late pachytene in the different *sun-1* phosphosite mutants. The durations of these three meiotic stages were measured by regarding SUN-1 aggregates and chromatin clustering as a readout for chromosome movement (see Material and Methods and Figure 3C). In the wild-type reference, the *sun-1(wt)*-expressing transgenic line, the TZ length comprised 21.4±5.0% of the meiotic part of the gonad. The TZ combined with the early pachytene zone was equally as long as the zone that comprised mainly nuclei in the mid/late pachytene stage (Figure 3D, exact quantifications in Table S2). Germlines expressing *sun-1* phosphosite mutants (glutamic acid and alanine substitutions) exhibited only moderately altered ratios between the TZ and early pachytene. Mutants expressing the multiple glutamic acid–substitution line *sun-1(6E)*, which mimics constitutive SUN-1 phosphorylation, showed the most pronounced prolongation of the TZ (Figure 3D and Table S2). Thus, glutamic acid substitutions on SUN-1 elicited only a mild meiotic delay at the stage of chromosome movement. These data were surprising, since we previously observed stronger phenotypes in *sun-1* phosphosite substitution lines [14] generated by particle bombardment; the phenotypes observed in these lines were mainly caused by *sun-1* dosage reduction due to limitations of the bombardment transformation technique (see Discussion).

Formation of the synaptonemal complex (unpublished data) and meiotic DSB repair kinetics, followed by RAD-51 foci quantification [23], [24] (Figure 3E), were not substantially altered compared to the wild type, except for in one *sun-1* phosphosite mutant. In *sun-1(allA)*-expressing germlines, in which all of the phosphosites were replaced with unphosphorylatable alanine, full elongation of the SC was strongly delayed, but ultimately achieved, since all chromosomal axes colocalized with SYP-1 in late pachytene. In early pachytene, *sun-1(allA)* displayed strongly reduced synapsis (Figure 3F). This delay in SC formation may also explain the accumulation of RAD-51-marked recombination intermediates in the mid-pachytene region that we observed in *sun-1(allA)-*expressing germlines (Figure 3E). A delay in homologous pairing does not account for the delay in synapsis, since the kinetics of homologous pairing were not affected, as assessed by fluorescence in situ hybridization (FISH) (Figure S3A).

Since SUN-1 phosphorylation was prolonged upon irradiation (Figure 1E and 1D), we tested whether offspring quality was impaired in *sun-1(allA)* mutants after exposure to 90 Gy of ionizing radiation (Table S3). *sun-1(allA)* mutants displayed decreased offspring viability after ionizing radiation compared to the wild type. Embryonic lethality in *sun-1(wt)* was most pronounced 0–24 h post-irradiation when the meiocytes giving rise to the dead embryos were in pachytene or later stages at the time of irradiation. By comparison, 24–48 h post-irradiation, the number of viable offspring was increased in the wild type. The meiocytes giving rise to these embryos were in the TZ or early pachytene stage at the time of irradiation. As shown above, these are the nuclei that were able to maintain SUN-1 phosphorylation in response to massive DNA damage while migrating down the gonadal tube. In contrast, offspring quality was not increased at this later time point post-irradiation in *sun-1(allA)* mutants (Table S3). These results suggest that SUN-1 phosphorylation contributes to tolerance against ionizing radiation.

Overall, the *sun-1* phosphosite mutants displayed only subtle meiotic defects. Glutamic acid substitution attempting to mimic constitutive phosphorylation of SUN-1 was neither capable of delaying the onset of mid/late pachytene, nor was it capable of prolonging the persistence of SUN-1 aggregate(s) to an extent similar to that observed in meiotic mutants (synapsis- or recombination-defective mutants). Defects in SUN-1 aggregate morphology became apparent only when SUN-1 was rendered entirely unphosphorylatable. Strikingly, the ability of SUN-1 to undergo phosphomodification increased the tolerance for ionizing radiation (this was most effective for nuclei that were in TZ/early pachytene when exposed to the damage). Furthermore, phosphomodification was required for SC formation with wild-type kinetics.

### SUN-1 phosphorylation is required for aggregate persistence and chromosome mobility beyond the TZ

**Figure 4:**
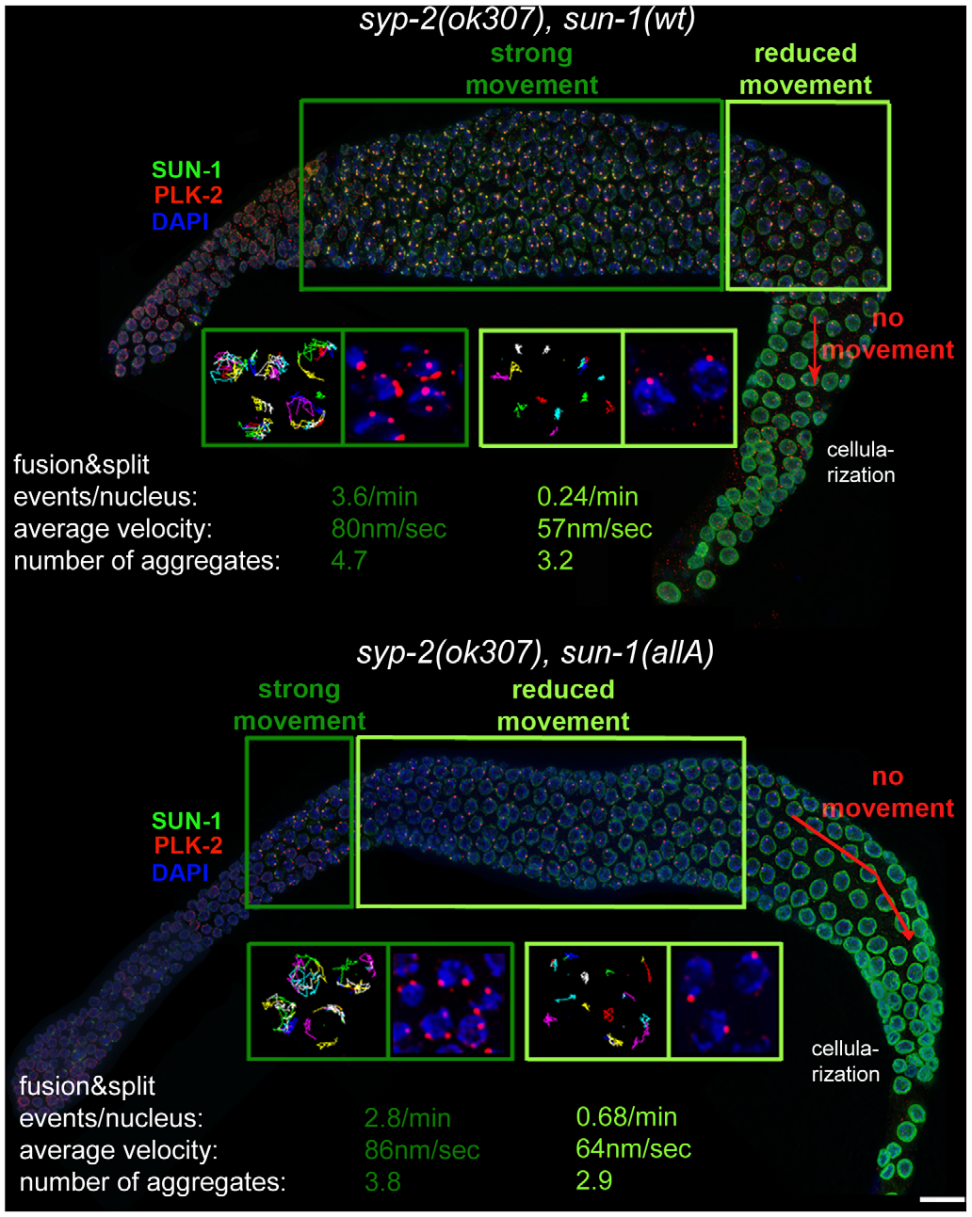
Effect of nonphosphorylatable SUN-1 on aggregate persistence, PLK-2 localization, and chromosome mobility beyond TZ. Hermaphrodite gonad of *sun-1(wt); sun-1(ok1282), syp-2(ok307)* (top) and *sun-1(allA); sun-1(ok1282); syp-2(ok307)* (bottom) stained with DAPI (blue), anti-GFP to highlight SUN-1 (green), and anti-PLK-2 (red). Dark green and light green frames highlight zones with distinct chromosome movement patterns: magnifications of nuclei in the corresponding zones below, showing displacement tracks of 2D plotted chromosome end movements over 3 min (left) and PLK-2 (red) and DAPI (blue). Bottom: average number of aggregates, aggregate velocity (in nm/sec), and fusion/split events (per nucleus/min) for nuclei from corresponding zones. Scale bar, 10 µm.

Given the fact that *sun-1(allA)* mutants showed a severe delay in SC completion, we expected a greater extension of TZ-like chromosome movement and clustered chromatin. However, *sun-1(allA)* prolonged the TZ characteristics only slightly. We therefore crossed the *sun-1(allA)* mutation into a synapsis mutant to assess whether the meiotic progression delay normally observed in the synapsis mutants was influenced by the SUN-1 phosphorylation status. In *sun-1(wt); syp-2(ok307)* mutant germlines, the TZ length comprised 79.1±2.1% of the meiotic part of the gonad, and the mid/late pachytene zone was reduced to 18.1±3.8%. By comparison, in wild-type worms, this region would account for 48.9±6.4% of the meiotic gonad (Figure 3A and Table S1). We found that *sun-1(allA)* expression in *syp-2* mutants restored the length of the shortened pachytene observed in *sun-1(wt); syp-2* mutants to 41.3±5.9% of the meiotic gonad (Figure 3D and Table S2). SUN-1 aggregates dissolved prematurely and chromatin appeared to be relaxed earlier than in the *sun-1(wt); syp-2* reference strain (Figure 4A and Figure S4A). No other phosphosite mutant was able to reduce the TZ length in *syp-2* mutants (Figure 3D and Table S2).

In *plk-2(ok1936); syp-1(me17)* mutant germlines, prolonged chromosome movement and asynapsis-triggered apoptosis were abolished [12]. Because PLK-2 is the proposed kinase for at least SUN-1 S12 [11], we wanted to test whether *sun-1(allA)* was a mediator for synapsis-dependent apoptosis. Unlike the *plk-2* deletion, expression of *sun-1(allA)* was not able to reduce the elevated numbers of apoptotic corpses (visualized by SYTO 12) in *syp-2* mutants; therefore, SUN-1 is not the responsible target for *plk-2* with respect to apoptosis induction. Consistent with this observation, the number of apoptotic corpses did not increase in *sun-1(6E)* germlines micking constitutive phosphorylation (Table S4).

DSB turnover, as judged by staining for RAD-51, did not differ between *syp-2; sun-1(wt)* and *syp-2* combined with any SUN-1 phosphosite mutant. RAD-51 foci accumulated in late pachytene and disappeared before the diakinesis stage (Figure S4 and unpublished data). These results support the idea that the observed accumulation of RAD-51 foci in *sun-1(allA)* mutants (Figure 3E) was due to a delay in repair and not due to an increase in DSB formation. Most likely, this delay in repair is a consequence of the delayed SC formation in *sun-1(allA)* mutants (Figure 3F).

*sun-1(allA)* decreased the length of the zone displaying more than one SUN-1 aggregate in *syp*-2 mutants to 48.7±5.3% of the meiotic gonad (Table S2). Chromatin also appeared to be more relaxed in early pachytene in *sun-1(allA) syp-2* mutants. *In vivo* time lapse imaging and 2D plotting of aggregate movement in *syp-2; sun-1(wt)* showed that over almost the entire gonad, aggregates displayed similar relative movement towards each other and similar numbers of splitting and fusion events (Figure 4 and [30]). The movements of chromosome ends towards each other and splitting and fusion events were strongly reduced only in the last 3–5 cell rows, before the SUN-1 aggregates disappeared (the region where nuclei start to leave the syncytium). Long, directional displacement tracks were strongly diminished. Aggregates in this region of the *syp-2* gonad did not move over longer distances, but were rather pushed and pulled on the spot; aggregate velocity by itself was not reduced in these nuclei (Figure 4). In contrast, aggregate dynamics in *syp-2; sun-1(allA)* were markedly different from those in *syp-2; sun-1(wt)*. Most of the aggregates from nuclei in the region corresponding to pachytene displayed restricted movement. Splitting/fusion events and long displacement tracks were absent. The properties of movement resembled those of aggregates in *syp-2; sun-1(wt)* at the very end of the prolonged TZ (Figure 4). Only aggregates at the beginning of the meiotic gonad, corresponding to early TZ in the wild type, displayed the highly dynamic wild-type aggregate behavior, with characteristic long displacement tracks and splitting and fusion events (Figure 4). We therefore conclude that the SUN-1 phosphomodifications are required to keep chromosomes moving continuously until full synapsis is reached.

Localization of the ZIMs to the pairing centers at the nuclear envelope is required for SUN-1 aggregate formation, chromosome movement, and PLK-2 localization [11], [12]. Therefore, we tested whether ZIM localization was affected in *syp-2; sun-1(allA)–*expressing germlines by following ZIM-3, the pairing-center binding protein for chromosomes I and IV [8]. ZIM-3 was present at the nuclear envelope, and colocalized with SUN-1 aggregates throughout the gonad in *syp-2; sun-1(wt)* ([14] and Figure S4B); a similar pattern was observed in *syp-2; sun-1(allA)–*expressing lines. Therefore, instability of ZIM localization at the nuclear envelope cannot account for the premature reduction in chromosome movement and SUN-1 aggregate dissolution observed in syp-2; *sun-1(allA)*.

Features of *sun-1(allA); syp-2* are to some extent analogous to the *plk-2(ok1936); syp-1* phenotype [12], in which SUN-1 aggregates form and chromosomes initially move. Despite the synapsis deficiency in both *sun-1(allA); syp-2* and *plk-2(ok1936); syp-1* mutants, chromosome movement stops prematurely. To gain more mechanistic insight into how SUN-1 phosphorylation mediates *plk-2*-dependent leptotene/zygotene arrest, and why *sun-1(allA)* partially, but not completely, phenocopies the *plk-2* deletion, we analyzed PLK-2 localization in the *syp-2; sun-1(allA) and syp-2; sun-1(wt)–*expressing lines. In *syp-2; sun-1(wt)*, multiple PLK-2 foci per nucleus localized to all movement-competent SUN-1 aggregates over almost the entire length of the gonad. Only in the last cell rows of the meiotic gonad, where there was reduced movement, did PLK-2 localization diminish; most of the time, only one PLK-2 focus per nucleus was visible (Figure 4). In contrast, PLK-2 localized to SUN-1 aggregates only in the first cell rows after meiotic entry in *syp-2; sun-1(allA)*–expressing germlines. Analogous to the smaller nonphosphorylatable aggregates formed in *syp-2; sun-1(allA)*–expressing germlines, PLK-2 signals were much weaker in *sun-1(allA)* germlines from the beginning of the TZ onwards (Figure 4 and unpublished data). In the remainder of the gonad, PLK-2 signals were strongly decreased or absent at the autosomal pairing centers and only persisted at the X-chromosomal pairing center in *syp-2; sun-1(allA)* (Figure 4 and unpublished data).

These results demonstrate that SUN-1 phosphorylation is required, not only for SUN-1 aggregate stability, but also for robust localization of PLK-2 to the autosomal pairing centers. The consequences of this become most apparent under challenging conditions. Because PLK-2 interacts with SUN-1 and phosphorylates it and, in return, depends on SUN-1 phosphomodification to localize to chromosome end attachments, we propose the existence of a self-reinforcing feedback loop between PLK-2 localization and SUN-1 phosphorylation at chromosomal attachment sites at the nuclear envelope. Under challenging conditions, this feedback loop maintains PLK-2 at the nuclear envelope and thereby allows chromosome mobility. Therefore, the delay in meiotic progression observed in synapsis mutants is dependent on SUN-1 phosphorylation.

### SUN-1 S12 is not phosphorylated at the nuclear envelope attachment site of the constitutive lone X chromosome in males

**Figure 5:**
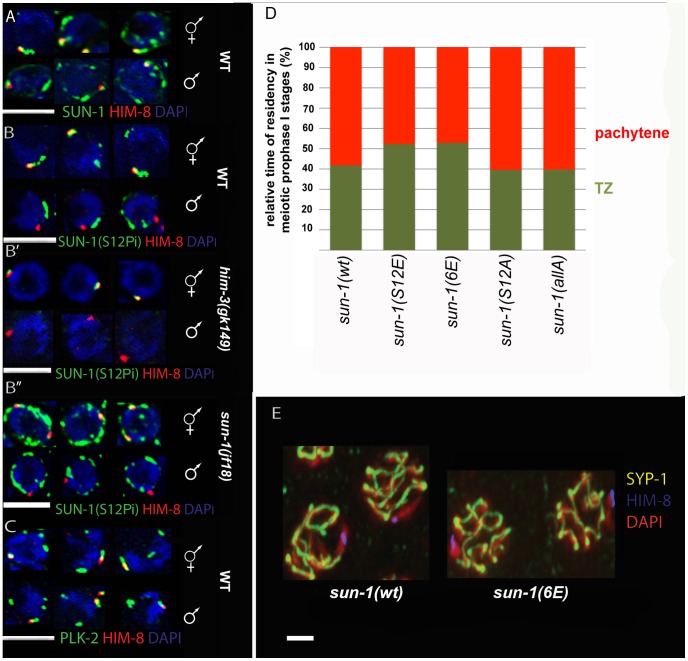
SUN-1 S12 phosphorylation in male meiosis. (A) Representative TZ nuclei of a *sun-1(wt)* hermaphrodite gonad (upper panel) and a *sun-1(wt)* male gonad (lower panel) to highlight SUN-1 (anti-GFP, green), anti-Him-8 (red), and DAPI (blue). (B) Representative nuclei from wild-type hermaphrodite TZ (upper panel) and wild-type male TZ (lower panel) stained with anti-SUN-1 S12Pi (green), anti-HIM-8 (red), and DAPI (blue). (B′) Representative nuclei from *him-3 (gk149)* hermaphrodite TZ (upper panel) and *him-3 (gk149)* male TZ (lower panel) stained with anti-SUN-1 S12 Pi (green), anti-HIM-8 (red), and DAPI (blue). (B″) Representative nuclei from *sun-1(jf18)* hermaphrodite TZ (upper panel) and *sun-1(jf18)* male TZ (lower panel) stained with anti-SUN-1 S12 Pi (green), anti-HIM-8 (red), and DAPI (blue). (C) Representative wild-type TZ nuclei of a hermaphrodite gonad (upper panel) and a male gonad (lower panel) costained with anti-PLK-2 (green), anti-Him-8 (red), and DAPI (blue). (D) Relative time of residency in different meiotic stages in different *sun-1* phosphorylation mutant backgrounds in males, assessed by presence of SUN-1 aggregates; zones were normalized to the individual length of the scored gonad (from meiotic entry to diplotene). Nuclei were sorted into two categories: “TZ” (dark green, more than one SUN-1 aggregate) and “pachytene” (red, no SUN-1 aggregates). (E) Pachytene zone nuclei of *sun-1(wt)* and *sun-1(6E)* stained with anti-HIM-8 (blue), anti-SYP-1 (yellow), and DAPI (red). Note that the single male X chromosome was unsynapsed in both WT and *6xE* substitution lines.

*C. elegans* hermaphrodites have the karyotype X/X, whereas males arise by spontaneous nondisjunction of the X chromosome and display the X/O karyotype. The lone X chromosome is therefore unable to undergo pairing and synapsis with a homologous partner. While in hermaphrodites, any unsynapsed chromosome would trigger a cell cycle delay [21], the failure of the single X chromosome to undergo synapsis in *C. elegans* males does not activate the meiotic checkpoint (whereas asynapsis of autosomes does). Therefore, the single X chromosome in males must have adapted a mechanism that shields it from the synapsis checkpoint [28]. Nevertheless, in males, the lone X-chromosomal pairing center induces, as in hermaphrodites, a SUN-1 aggregate. In both sexes, HIM-8 always colocalizes with a SUN-1 aggregate (Figure 5A).

SUN-1 phosphorylation at S12 exclusively occurs on SUN-1 molecules present in aggregates adjacent to individual chromosomes. Therefore, we analyzed S12 phosphorylation at the X-chromosomal SUN-1 aggregate in the male germline. The single male X chromosome never colocalized with a SUN-1-phosphorylated S12 signal, despite the presence of phosphorylated S12 signals colocalizing with the autosomes (Figure 5B). In contrast, the paired and unpaired X-chromosomal signals in hermaphrodites consistently colocalized with the S12 phospho-signal (Figure 5B′ and 5B″).

Immunofluorescence analysis in male compared to hermaphrodite gonads is more challenging due to higher staining backgrounds in males. Assessing SUN-1 S12 phosphorylation on HIM-8-induced aggregates in *sun-1(jf18)* and *him-3(gk149)* provided further evidence that SUN-1 aggregates at the male X chromosome were excluded from phosphorylation at S12 (Figure 5B′ and Figure 5B″). In both cases, the lone X chromosome can be more easily distinguished from the autosomes. In *sun-1(jf18)*, SUN-1 aggregates do not collide [15], and in *him-3(gk149*), only the X-chromosomal aggregate forms, whereas the autosomal aggregates are absent [14], [36].

In contrast to S12, other phosphosites (S8, S24, and S43) displayed phosphorylation at the X-chromosome attachment site in males, equivalent to what was observed in hermaphrodites (unpublished data). Also in males, PLK-2 colocalized with all SUN-1 aggregates, including those induced by HIM-8 (Figure 5C). Thus, it appears that the lack of SUN-1 phosphorylated S12 on the male X chromosome was not due to a defect in recruiting PLK-2, the proposed kinase for S12.

We wanted to mimic constitutive phosphorylation on SUN-1 S12 to test if this would lead to the triggering of a progression delay by the lone X chromosome in males. We therefore measured the duration of leptotene/zygotene (TZ) and pachytene in the gonads of different *sun-1* phosphosite mutant males. We found that the average TZ length in the glutamic acid substitution lines *sun-1(S12E)* and *sun-1(6E)*) was slightly prolonged. The alanine substitution mutants *sun-1(S12A)* and *sun-1(allA)* did not have an altered TZ length (Figure 5D and Table S5). Therefore, mimicking constitutive SUN-1 phosphorylation at the normally nonphosphorylated X-chromosomal aggregate is not sufficient to evoke a full delay in progression, as seen for unsynapsed autosomes in males [28].

Asynapsis of one chromosome can affect the segregation of other chromosomes. This phenomenon can be observed in *zim-2* deletion mutants, which are incapable of pairing and synapsing of chromosome V, and which show increased levels of X-chromosomal nondisjunction [8]. The single unsynapsed X chromosome in males, however, does not seem to affect the segregation of the autosomes [8]. This fact and the observation that SUN-1 modifications are required for wild-type kinetics of SC assembly led us to ask whether keeping the SUN-1 aggregate unphosphorylated at the constitutively unsynapsed X chromosome would inhibit synapsis with a nonhomologous chromosome by slowing down its ability to synapse. We therefore assessed synapsis in *sun-1(6E)* males. As depicted in Figure 5E, similar to what was observed in wild-type animals, the single X chromosome was not observed to be engaged in synapsis with other autosomes in pachytene of *sun-1(6E)* males when costained for HIM-8 and SYP-1.

Based on the results above, we can conclude that in males, the constitutive lone X-chromosomal pairing center induced a SUN-1 aggregate, but this aggregate was not phosphorylated on S12, although the required kinase, PLK-2, was properly localized to the X-chromosomal pairing center and the X chromosome was mobile (unpublished data). This is consistent with the interpretation that SUN-1 phosphorylation represents an integral part of a checkpoint system that monitors successful achievement of meiotic tasks. Glutamic acid substitution of SUN-1 phosphosites failed to induce a complete synapsis checkpoint response. This suggests that the checkpoint response might be supported by additional factors, or that glutamic acid substitutions do not sufficiently mimic phosphorylation.

## Discussion

Phosphomodifications of the nuclear amino terminus of Matefin/SUN-1 and its concentration into aggregates at chromosome nuclear envelope attachment sites correlate with the presence of uncompleted meiotic tasks (unsynapsed chromosome axes, unrepaired DSBs, and an absence of certain crossover intermediates) in wild-type and mutant germlines. SUN-1 phosphomodifications are required to initiate a meiotic progression delay, and in this study, we provide evidence that they mediate a feedback loop involving SUN-1 aggregate stability and PLK-2 localization. This mechanism sustains leptotene/zygotene-characteristic chromosome mobility under challenging conditions, such as asynapsis, and is also required to synapse homologous chromosomes with wild-type kinetics.

Phosphorylation of SUN-1 is dispensable for chromosome pairing, loading, and maintenance of pairing center proteins at the nuclear envelope, and for induction of SUN-1 aggregates. However, the nonphosphorylatable SUN-1 aggregates are smaller and not maintained under challenging conditions. Furthermore, these smaller, unstable SUN-1 aggregates fail to maintain the colocalization of PLK-2 on pairing centers of autosomes beyond zygotene. Pairing center proteins remain at the nuclear envelope; nevertheless, directed movement of chromosome ends beyond zygotene is absent in nonphosphorylatable *sun-1* mutants. Furthermore, we demonstrate that this proposed meiotic surveillance mechanism is required for full wild-type offspring viability under challenging conditions. We observed that only nuclei in leptotene/zygotene are susceptible to SUN-1 modifications, and we therefore propose that this particular surveillance mechanism cannot be reinstalled later than zygotene (TZ).

### SUN-1 phosphomodification as part of a checkpoint that monitors incomplete meiotic tasks

Meiocytes enter meiosis in leptotene with unpaired and unsynapsed chromosome axes. In the TZ, DSBs are generated to give rise to the vital crossover between homologous chromosomes. Our results show that SUN-1 phosphorylation is sustained until chromosome axes are synapsed and DSB repair progresses beyond a certain stage. Interestingly, in the absence of DSBs in *spo-11* germlines, SUN-1 phosphorylation also persists, suggesting that crossover recombination needs to progress to a certain stage to permit SUN-1 dephosphorylation. SUN-1 phosphorylation correlates with ATM/ATL-dependent phosphorylation of CHK-1. The prolonged phosphorylation observed in *spo-11* mutants can be mitigated by treatment with low doses of ionizing radiation that are sufficient to rescue bivalent formation, but too low to induce checkpoint activation in all nuclei. This suggests that damage signals must be emanating from fully synapsed pairs of homologous chromosomes that have not yet formed a certain crossover intermediate. Independently, SUN-1 phosphorylation is sustained in synapsis-defective mutants in leptotene/zygotene. These results suggest that multiple checkpoint/DNA-damage signaling events lead to prolonged SUN-1 phosphorylation, consistent with the observation that individual loss of any of the known checkpoint players (*atm-1*, *atl-1*, *hus-1*, *cep-1*, and *pch-2*) does not reduce SUN-1 phosphomodification or the time spent in leptotene/zygotene (unpublished data). Furthermore we propose that it is not only meiotically induced DSBs that elicit damage signaling in leptotene/zygotene since CHK-1 appears activated even in the absence of *spo-11*. Therefore, the modifications are part of a surveillance machinery that monitors ongoing meiotic tasks. In contrast to synapsis-defective mutants, however, constitutive SUN-1 phosphorylation was not sufficient to fully arrest meiocytes in leptotene/zygotene, suggesting that other components participate in executing the meiotic arrest.

### Role of SUN-1 phosphorylation in responding to meiotic failure in prophase I progression

In nonphosphorylatable *sun-1* mutants, less PLK-2 was recruited to pairing centers at nuclear envelope attachment sites. Furthermore, PLK-2 was not stably maintained at autosomal pairing centers in a synapsis-defective mutant, despite the fact that the pairing center proteins were appropriately localized to these pairing centers. The depletion of PLK-2 at pairing centers correlates with restricted movement of chromosomal ends under challenging conditions. Under these conditions, in nonphosphorylatable *sun-1* mutants, wild-type-like movement occurred only in the first rows of TZ, thereby resembling the *plk-2* deletion mutant [11], [12]. We speculate that another kinase is sufficient to activate the key substrates for chromosome end–led movement, but for later stages (late zygotene/early pachytene), movement becomes dependent on *sun-1*-mediated PLK-2 recruitment.

PLK-1 can partially substitute for PLK-2, leading to incomplete homolog pairing and synapsis in the *plk-2* deletion mutant; in this case, the TZ is shortened and chromatin clustering in a synapsis mutant cannot be maintained [11], [12]. Nonphosphorylatable SUN-1, which is unable to maintain PLK-2 on chromosomal attachment sites, displays defects similar to those seen in the *plk-2* deletion mutant with regard to synapsis and chromatin clustering.

Consequently, we propose that phosphorylation of SUN-1 may be part of a self-reinforcing feedback loop between PLK-2-dependent SUN-1 phosphomodification and SUN-1 phosphorylation–dependent PLK-2 localization; this loop remains activated upon detection of unfinished meiotic tasks. After meiotic entry, meiotic chromosomal axes are defined by specific components, such as HTP-1-3 and HIM-3 [37]–[39]. Formation of meiotic axes is independent of CHK-2 [37]. We speculate that unsynapsed axes emit a *chk-2*-dependent signal that results in the phosphorylation of SUN-1 on several residues (in particular, serines 8, 24, and 43) and loading of the pairing center–binding proteins onto their respective pairing centers, which then recruit PLK-2. This ultimately results in the induction of SUN-1 aggregates, SUN-1 phosphorylation on serine 12 by PLK-2, and transmission of cytoplasmic kinetic forces to pairing centers [11], [12]. PLK-2-dependent modification of the pairing center–binding proteins and other unknown targets may then lead to directed movement of chromosome ends [12]. We speculate that phosphorylation of SUN-1 increases its ability to bind PLK-2 to stabilize it at the pairing centers, where PLK-2 reinforces the stability of SUN-1 aggregates and supports directed movement of chromosome ends until certain meiotic tasks are accomplished. Consistent with this model, PLK-2 was found in a protein complex with SUN-1 [11].

### Role of SUN-1 phosphorylation during synapsis

SUN-1 phosphorylation is required for wild-type kinetics of SC polymerization, but it is dispensable for pairing. Several interpretations of this result are possible. PLK-2 is required for a fully developed SC that is likely to assemble in a processive manner from the pairing centers [11], [12]. In leptotene/zygotene, PLK-2 localizes to SUN-1 aggregates, and from there it seems to be transferred onto synapsed parts of the chromosomes (Figure S1). In nonphosphorylatable SUN-1 aggregates, PLK-2 localization is unstable. Therefore, it is possible that the amount of PLK-2 that can effuse from the pairing center downwards along the axes is insufficient to fulfill its function there. However, we cannot exclude a direct involvement of SUN-1 phosphorylation in SC progression. Alternatively, we could speculate that smaller/weaker SUN-1 aggregates in the nonphosphorylatable background transmit less of the kinetic forces that are required to drive SC formation with wild-type kinetics between homologous chromosomes. Chromosome end–led movements have frequently been suggested not only to contribute to the search for the homologous partner, but also to play a role in resolving topological entanglements, interlocks, and unwanted interactions that might impede successful recombinational repair and SC formation [16]. Furthermore, chromosome motions might even provide the energy for recombinational repair [40]. Therefore, we propose that the coupling of SUN-1-mediated progression delay with chromosome movement is an elegant means to ensure chromosome motion until a crossover has been established on each chromosome.

### Reduction of *sun-1* gene dosage alters meiotic behavior

In a previous study, we observed stronger phenotypes in a *sun-1* substitution line generated by particle bombardment (biolistic transformation) compared to the phenotypes observed here with Mos single-copy insertion lines. In phosphomimetic mutants, the TZ was prolonged and SUN-1 aggregates persisted more dramatically. We also reported previously a much stronger accumulation and delayed disappearance of RAD-51 foci and greater meiotic chromosome mis-segregation [14]. It is likely that these lines were not single-copy lines and were subjected to germline silencing, because over generations, the GFP signals of the transgenes and the fertility of these lines decreased (the novel lines presented here did not show silencing over >20 generations). RNAi depletion of *sun-1* in a new Mos-generated line phenocopied the defects seen in the biolistically generated lines, suggesting that the previously reported phenotypes were due to incomplete rescue. SUN-1 aggregates and tightly clustered chromatin persisted and RAD-51 foci accumulated. These phenotypes, which resembled those of the lines generated by biolistic transformation, were more frequently induced by feeding SUN-1 RNAi to *sun-1(S12E)* and *sun-1(6E)* lines, but could also be observed when feeding SUN-1 RNAi to wild-type (*sun-1(wt)*) control animals. These phenotypes correlated with a reduction in SUN-1 and therefore offer an explanation for the differences observed when using transgenic lines that were generated by different methods (Figure S5B). Moreover, a homologous pairing defect, as previously reported for one of the substitution lines [14], could be induced when even more SUN-1 protein was depleted (Figure S5A).

### Male *C. elegans*: An example of the biological relevance of the SUN-1–mediated checkpoint

*C. elegans* males have a single X chromosome, and its unpaired/unsynapsed status does not lead to a progression delay. Therefore, it must be hidden from, or refractory to, the checkpoint machinery. At the nuclear envelope–associated end of the lone male X chromosome, SUN-1 is not phosphorylated at S12; nevertheless, it is mobile (unpublished data). This is consistent with a model in which SUN-1 phosphorylation installs a checkpoint mechanism that is competent to delay meiotic progression. We propose that an absence of this phosphorylation may be part of the mechanism to hide the single X from the checkpoint machinery that responds to unsynapsed chromosomes, which, in turn, precludes the checkpoint machinery from responding to signals emanating from the unsynapsed X. It will be interesting to learn whether the repressive chromatin environment of the single X chromosome [41] in males acts on PLK-2 activity, or if an as-yet unknown phosphatase “overrides” PLK-2 kinase activity at the X chromosome.

### A G2 meiotic checkpoint

In this study, we present evidence for the existence of a meiotic prophase checkpoint system at the nuclear envelope that responds to unfinished meiotic tasks, such as incomplete synapsis or recombination, by delaying meiotic progression. Most importantly, the SUN-1-mediated “checkpoint” system can only integrate a signal when the cell is still in a responsive state (zygotene and early pachytene), and long before the G2/M transition. We observed that nuclei that had progressed beyond early pachytene were unable to reestablish SUN-1 and CHK-1 phosphorylation. Recently, it was shown that a DNA-damage checkpoint at the G2/M transition was only responsive to very high doses of the DNA-damaging agent etoposide in mouse oocytes [42]. Moderate doses led to activation of DNA-damage responses, such as production of gamma H2AX chromatin, but ATM signaling and subsequent CHK1 signaling were not enough to elicit an efficient response. The potential surveillance mechanism for unfinished meiotic tasks proposed in our study (provided it also exists in vertebrates) may have been undetected by Marangos and Caroll because, in their study, oocytes were exposed to DNA-damaging agents past the stage of meiotic prophase in which SUN-1 phosphorylation is observed in *C. elegans*. Recently, vertebrate Sun1^−/−^ Sun2^−/−^ embryonic fibroblasts were shown to accumulate DNA damage more rapidly and failed to arrest cell cycle progression in response to certain genotoxic agents [43]. This suggests a potentially conserved role for SUN-domain proteins in the DNA-damage response. Because leptotene/zygotene bouquet formation (SUN/KASH-dependent chromosome end attachment with prominent Sun protein relocation and concentration into chromosome ends) is conserved in mammals [44], [45], it will be worthwhile in future investigations to determine whether mammalian oocytes have adapted a similar checkpoint mechanism to slow down progression, leading to prolonged letotene/zygotene chromosome movement when unfinished meiotic tasks are detected.

## Materials and Methods

### Genetics

Maintenance and cultivation of worms were based on standard protocols [46]. Following strains were used in this study:

N2 (Bristol), *ced-3(n717)* [*47*] *, syp-2(ok307)*/nT1[qIs51](IV:V) [24], VC1873 *rad-51(ok2218)*/nT1[qIs51](IV:V), *sun-1(jf18)*/nT1[qIs51](IV:V) [15], *him-3(gk149)*/nT1[qIs51](IV:V) [36], *spo-11(ok79)*/nT1[qIs51] (IV:V) [34], EG4322 *(ttTi5605* II;*unc-119(ed3)*II*)* [*35*], UV96 *atm-1(gk186)* I; *atl-1(tm853)* IV/nT1[qIs51] (IV;V) [14].

Following strains were generated using the MosSCI system [35] in this study: UV29 *sun-1(ok1282)V/*nT1[qIs51](IV;V), *ttTi5605 jfSi1[Psun-1::GFP cb-unc-119(+)]*II, UV31 *sun-1(ok1282)V/*nT1[qIs51](IV;V), *ttTi5605 jfSi3 [Psun-1S12E::GFP cb-unc-119(+)*]II, UV88 *sun-1(ok1282)*V/nT1[qIs51](IV;V), *ttTi5605 jfSi23 [Psun-1(S8, 12, 24, 43, 58, 62E)::GFP cb-unc-119(+)]*II, UV87 *sun-1(ok1282)*V/nT1[qIs51](IV;V), *ttTi5605 jfSi4 [Psun-1(12A)::GFP cb-unc-119(+)]*II, UV68 *sun-1(ok1282)*V/nT1 [qIs51](IV;V), *ttTi5605 jfSi9 [Psun-1(8, 12, 24, 36, 43, 58, 62A)::GFP cb-unc-119(+)]*II.

Following genotypes were generated during this study:

UV93 *syp-2(ok307)V/*nT1[qIs51](IV;V)*; sun-1(ok1282)V/*nT1[qIs51](IV;V), *ttTi5605 jfSi1[Psun-1::GFP cb-unc-119(+)]*II, UV90 *syp-2(ok307)*V/nT1 [qIs51](IV;V), *sun-1(ok1282)V/*nT1[qIs51](IV;V), *ttTi5605 jfSi3 [Psun-1S12E::GFP cb-unc-119(+)*]II, UV92 *syp-2(ok307)V/*nT1[qIs51](IV;V); *sun-1(ok1282)*V/nT1[qIs51](IV;V), *ttTi5605 jfSi23[Psun-1(S8, 12, 24, 43, 58, 62E)::GFP cb-unc-119(+)]*II, UV91 *syp-2(ok307)V/*nT1[qIs51](IV;V), *sun-1(ok1282)*V/nT1[qIs51](IV;V), *ttTi5605 jfSi4[Psun-1(12A)::GFP cb-unc-119(+)]*II, UV86 *syp-2(ok307)*V/nT1[qIs51](IV;V); *sun-1(ok1282)V/*nT1[qIs51](IV;V), *ttTi5605 jfSi9[Psun-1(8, 12, 24, 36, 43, 58, 62A)::GFP cb-unc-119(+)]*II.

### Phosphosite mutants

SUN-1 Pi correlates with SUN-1 aggregate formation and chromosome movement. But phosphorylations and aggregate formation do not depend on movement or pairing ([11]–[14]). We therefore asked whether SUN-1 phosphorylation was required for SUN-1 aggregate formation and chromosome movement resulting in chromatin clustering and homologous pairing ([11], [12], [14]) or even to arrest nuclei in leptotene/zygotene as we suggested previously [14]. For this purpose we generated different SUN-1 phosphosite mutants by using the MosSCI system, which allows integration of a transgene at a defined locus in the genome as a single copy [35]. We generated five different mutant transgenic *sun-1* lines, all C-terminally tagged with GFP and crossed them to the *sun-1(ok1282)* deletion background. The wild type control (referred to as *sun-1(wt)*) was compared to two different glutamic acid substitution lines, referred to as *sun-1(S12E)* and *sun-1(6E)*, in which all six serines doubtlessly identified as target sites were exchanged to glutamic acid (mass spec analysis could not differentiate between S35 and T36). In addition the wild type control was compared to two non-phosphorylatable lines (referred to as *sun-1(S12A)* and *sun-1(allA)*, in which all residues found to be phosphorylated, including T36, were replaced by alanine). Subjecting this latter line to mass spectrometry analysis did not detect residual SUN-1 phospho-signals (data not shown).

### Cytological preparations and immunofluorescence analysis

Dissection and immunostaining was performed as described in [14]. When assessing the TZ length in males, male worms were dissected 36–48 h post L4 at 16°C.

Following antibodies were used in this study:

Rat anti-SUN-1 phospho-serine 43 (1∶1000) (antisera were produced against the following phospho-peptide: CVT RRD S(PO_3_H_2_)QP G), guinea pig anti-SUN-1 phospho-serine 12 (1∶1500) [14], rabbit anti-RAD-51 (1∶100) [24], rabbit anti-SYP-1 (1∶200) [21], mouse anti-GFP (1∶500) (Roche Diagnostics), rabbit anti HIM-8 (1∶5000) (Novus), guinea pig anti HIM-8 (1∶500) [10], anti-ZIM-3 (1∶100) [14], guinea pig anti-SUN-1 phospho-serine 8 (1∶700) [14], rabbit anti-PLK-2 (1∶15) [11], anti HTP-3 [38], alexa fluor 568 goat anti-guinea pig (1∶500) (Invitrogen), anti-Chk1 (S345Pi) (1∶100) (Cell Signaling), alexa fluor 488 goat anti-guinea pig (1∶500) (Invitrogen), alexa fluor 568 goat anti-rabbit (1∶500) (Invitrogen), alexa fluor 488 goat anti-rabbit (1∶500) (Invitrogen), alexa fluor 488 goat anti-mouse (1∶500) (Invitrogen).

### Microscopic evaluation of fixed samples

A Zeiss Axioskop epifluorescence microscope was used in combination with a cooled CCD camera (Photometrics). 3D stacks of images were taken with MetaVue software (Universal Imaging), deconvolved with AutoDeblur software (AutoQuant Imaging) and projected with Helicon Focus software (Helicon Soft Ltd.).

Alternatively, a Deltavision deconvolution microscope and SoftWoRx image analysis deconvolution software system (both Applied Precision, Inc.) was used. Data analysis was performed using ImageJ (NIH).

### Assessment of meiotic prophase duration

To define the duration of meiotic prophase I stages with respect to chromosome movement and SUN-1 aggregate behavior we quantified how many nuclei per cell row in the gonadal tube from TZ to cellularization displayed more than one SUN-1 aggregate (paralleled by tightly clustered chromatin), one SUN-1 aggregate (paralleled by loosely clustered chromatin) or no SUN-1 aggregate (accompanied by chromatin distributed throughout the nuclear volume). If the majority of nuclei per cell row met one of the three criteria the cell row was scored as “TZ” (more than one aggregate), “early pachytene” (one aggregate) or “full pachytene” (no aggregate) (Figure 3C). The length of these zones was normalized to the shape and length of the meiotic gonad.

### Fluorescence in situ hybridization

FISH was performed as described in [15] using a PCR generated probe against the 5 S ribosomal locus.

### Irradiation assay

Hermaphrodites were exposed to ionizing irradiation from a _137_Cs source (90, 70 or 7.5 Gy). Dissection and cytological analysis were done 20 min, 90 min, 120 min, 8 h, or 27 h post-irradiation.

### Live imaging

Live imaging of SUN-1 aggregates was performed as described in [30]. Data analysis was performed using ImageJ (NIH) using the plugins “StackReg” and “Manual Tracking”.

### Mass spectrometry

Mass spectrometry was performed as described previously in [14] except for using the GFP-trap for immunoprecipitation (Chromotek). SUN-1::GFP purified from *sun-1(allA)* was subjected to mass spectrometry and no phosphorylation of S35 could be detected.

### RNA interference

RNAi feeding was performed as described in [48]. L1 to L4 larvae were pre-selected and incubated at 20°C until the animals reached 24 h post L4 stadium. Then gonads were dissected.

### Apoptosis quantification

L4 larvae were pre-selected and incubated for 24–36 hours at 20°C. Worms were collected into M9 solution containing 33 µM SYTO-12 (Molecular Probes) for 90 min in the dark. Worms were transferred to seeded plates for 30 min and then mounted on 2% agarose pads in 2 mM levamisole. The quantitative analysis was performed using a Leica DM6000 fluorescence microscope, Leica DC 350 FX camera under the control of Leica LAS AF 6000 software.

## Supporting Information

Figure S1 Colocalization of SUN-1 aggregates, SUN-1 phosphorylation signals, ZIMs, and PLK-2, as observed in this and previous studies [11], [12], [14], [30]. Representative wild-type hermaphrodite nuclei from TZ (left), early pachytene (middle), and late pachytene (right). All nuclei were stained with DAPI (blue). (A) SUN-1 (green) forms aggregates in the TZ; these aggregates are phosphorylated on SUN-1 S12 (red). SUN-1 not within aggregates is not phosphorylated on S12. In early pachytene, only one aggregate remains, and it is phosphorylated on S12. In late pachytene, aggregates and SUN-1 phosphorylation on S12 are gone. (B) Phosphorylation of SUN-1 S12 (red) and S43 (green) are seen at the same time. S43 phosphorylation pattern highlights the entire nuclear envelope, including the SUN-1 aggregates at chromosome ends. In early pachytene, SUN-1 S43 remains phosphorylated on the last aggregate (where it overlaps with S12 phosphorylation) and on the entire nuclear envelope. In late pachytene, S43 and S12 phosphorylation is gone. S43 phosphorylation pattern (green) overlaps with S24 (C, red) and S8 (D, red). (E) Autosomal pairing-center binding protein ZIM-3 (red) always colocalizes with SUN-1 (green) aggregates in the TZ. In early pachytene, ZIM-3 does not colocalize with the last remaining prominent SUN-1 aggregate. (F) HIM-8 (red), in contrast, remains colocalized with SUN-1 (green) aggregates in early pachytene. (G) PLK-2 (red) shares the localization pattern with its target phosphorylation site SUN-1 S12 (green) at SUN-1 aggregate(s) in the TZ and early pachytene. PLK-2 starts to localize to synapsing chromosomes from the TZ onwards and is found on all synapsed axes by late pachytene. (H) PLK-2 (red) and S43 phosphorylation (green) only overlap at SUN-1 aggregates. (I) Displacement tracks of SUN-1 aggregates represent 2D plotted chromosome end movements over 3 min. Scale bar, 2 µm.
(TIF)

Figure S2 Occasional chromatin clustering in pachytene correlates with synaptic failure. (A) Mid/late pachytene nuclei of a wild-type hermaphrodite gonad stained with anti-SYP-1 (left, green), anti-HTP-3 (left, red), anti-SUN1 S43Pi (right, red), and DAPI (right, blue). Nucleus in the middle with SUN1 phosphorylation has clustered chromatin and partly unsynapsed chromatin, in contrast to the surrounding nuclei with pachytene characteristics. Scale bar, 5 µm. (B) Nuclei with high numbers of RAD-51 foci, phosphorylated SUN-1, and clustered chromatin are also present in mid/late pachytene in apoptosis-deficient mutants. *ced-3(n717)* mutant hermaphrodite gonad stained with DAPI (top, blue in merged), anti-SUN-1 S43Pi (middle, green in merged), and anti-RAD-51 (bottom, red in merged). Arrow indicates nucleus in late pachytene zone with clustered chromatin, phosphorylated SUN-1, and abundant RAD-51 signal. (C) SUN-1 phosphorylation correlates with phosphorylated CHK-1 in wild type and mutants. WT (left) and *spo-11(ok79)* mutant (right) hermaphrodite gonad stained with DAPI (top, blue in merged), anti-SUN-1 S43Pi (middle, green in merged), and anti-CHK-1 S345Pi (bottom, red in merged). (D) CHK-1 phosphorylation depends on ATM/ATL. WT (top) and *atm-1(gk186); atl-1(tm853)* mid/late pachytene section; DAPI (blue) and antiCHK-1 S345Pi (red). (E) Irradiation-induced damage correlates with persistent SUN-1 phosphorylation. Pachytene WT hermaphrodite gonad dissected 27 h after 70 Gy gamma irradiation; anti-SUN-1 S12Pi (red), anti-RAD-51 (green), and DAPI (blue, right). Cells devoid of RAD-51 signal are also devoid of SUN-1 S12Pi signal (arrowheads). (F) Representative nuclei from TZ (left) early pachytene (middle) and mid/late pachytene (right) in *rad-51(ok2218)*. SUN-1 aggregates phosphorylated on S12 (red) colocalize with the HIM-8 signals (green) in all three zones. Scale bars: 5 µm in (A),10 µm in (B–E) and 2 µm in (F).
(TIF)

Figure S3 Homologous pairing and ZIM loading in a SUN-1 nonphosphorylatable mutant follow wild-type kinetics. (A) Pairing of chromosome V in *sun-1(allA); sun-1(ok1282)* mutant and *sun-1(wt); sun-1(ok1282)* was evaluated by FISH, highlighting 5S rDNA. Percentages of paired signals were assessed after dividing gonads into six zones of equal length. (B) *sun-1(wt); sun-1(ok1282)* and *sun-1(allA); sun-1(ok1282)* (bottom) hermaphrodite gonads stained with DAPI (top, blue in merged), anti-GFP (middle, green in merged), and anti-ZIM-3 (bottom, red in merged). Scale bar, 10 µm.
(TIF)

Figure S4 Nonphosphorylatable *sun-1* mutations do not affect DSB repair kinetics in a *syp-2* mutant. (A) *sun-1(wt); sun-1(ok1282); syp-2(ok307)* (left) and *sun-1(allA); sun-1(ok1282); syp-2(ok307)* (right) hermaphrodite gonads stained with DAPI (top, blue in merge), anti-GFP (middle, green in merge), and anti-RAD-51 (bottom, red in merge). (B) SUN-1 phosphorylation is dispensable for stable loading of ZIMs. *sun-1(wt); sun-1(ok1282); syp-2(ok307)* (left) and *sun-1(allA); sun-1(ok1282); syp-2(ok307)* (right) hermaphrodite gonads stained with DAPI (blue) and anti-ZIM-3 (red). Scale bars, 10 µm.
(TIF)

Figure S5 Decreased SUN-1 protein leads to failure of chromosome pairing. (A) Pachytene hermaphrodite *sun-1(wt); sun-1(ok1282)* gonads subjected to *sun-1* RNAi stained with DAPI (top, blue in merge), anti-GFP (middle, green in merge), and anti-HIM-8 (bottom, red in merge). (B) Reduction in SUN-1 protein dosage leads to prolonged SUN-1 aggregation and chromatin clustering. *sun-1(wt); sun-1(ok1282)* hermaphrodite gonads subjected to *sun-1* RNAi stained with DAPI (top, blue in merge) and anti-GFP (bottom, green in merge). Scale bar, 10 µm.
(TIF)

Table S1 SUN-1 aggregate behavior in TZ nuclei of *sun-1(allA)*. SUN-1::GFP aggregates recorded by *in vivo* time-lapse microscopy in TZ nuclei of *sun-1(wt)* and *sun-1(allA)* followed by 2D plotting for manual analysis. Duration, 3 min. Variations correspond to the standard deviations. Two-tailed *t*-test for fusion events, 0.107 and for splitting events, 0.103. n, represents number of independently tracked one minute sequences.
(DOCX)

Table S2 Relative duration of leptotene/zygotene, early pachytene, and middle/late pachytene in *sun-1* phosphosite mutants during oogenesis. Relative duration of meiotic stages were assessed by quantifying cell rows in the meiotic part of the gonad according to the following criteria: more than one SUN-1 aggregate (“TZ”), one or more SUN-1 aggregates (“aggregate zone”, TZ plus early pachytene), or no aggregates (“zone without aggregates”). When >50% of nuclei in a cell row met one of the three criteria, the cell row was scored as such. Percentages ± standard deviation shown represent numbers normalized to gonad length from meiotic entry to beginning of cellularization (diplotene stage in the wild type). *p<0.01 between the reference lines *sun-1(wt)* and *syp-2; sun-1(wt)* and the respective mutant lines in two-tailed *t*-test. n, number of gonads counted.
(DOCX)

Table S3 Number of viable offspring and hatch rate of nonphosphorylatable *sun-1* mutant. L4 hermaphrodites were irradiated (90 Gy) or not irradiated, and the viability of eggs between 2 and 48 h after irradiation was assessed at 20°C. *p<0.001 in a two-tailed t-test between *sun-1(wt)* and *sun-1(allA)*. n, number of animals evaluated.
(DOCX)

Table S4 Number of apoptotic corpses per gonad arm. Age-matched hermaphrodites were stained with SYTO 12. Positive nuclei were scored as apoptotic corpses. Variations indicate the standard error of the mean (SEM). Each genotype was analyzed in at least three independent experiments. *n*, number of scored gonad arms.
(DOCX)

Table S5 Relative duration of leptotene/zygotene (TZ) and pachytene in *sun-1* phosphosite mutants during male meiosis. Relative durations of meiotic stages were assessed by quantifying cell rows in the meiotic part of the gonad according to the following criteria: more than one SUN-1 aggregate (“TZ”) or no aggregates (“pachytene”). When >50% of nuclei in a cell row met these criteria, the cell row was scored as such. Percentages ± standard deviation shown represent numbers normalized to gonad length from meiotic entry to beginning of diplotene. n, number of gonads counted.
(DOCX)

Video S1 *In vivo* 2D-plotted time-lapse imaging of TZ nuclei of GFP-labeled *sun-1(wt); sun-1(ok1282)* hermaphrodite gonads. Frames recorded every 5 s (total recording time, 3 min).
(AVI)

Video S2 *In vivo* 2D-plotted time-lapse imaging of TZ nuclei of GFP-labeled *sun-1(allA); sun-1(ok1282)* hermaphrodite gonads. Frames recorded every 5 s (total recording time, 3 min).
(AVI)
