# Supplementary figures and images for: Matefin/SUN-1 Phosphorylation Is Part of a Surveillance Mechanism to Coordinate Chromosome Synapsis and Recombination with Meiotic Progression and Chromosome Movement
Source: PLoS Genet. 2013 Mar 7;9(3):e1003335. doi: 10.1371/journal.pgen.1003335 (PMC3591285; doi:10.1371/journal.pgen.1003335)

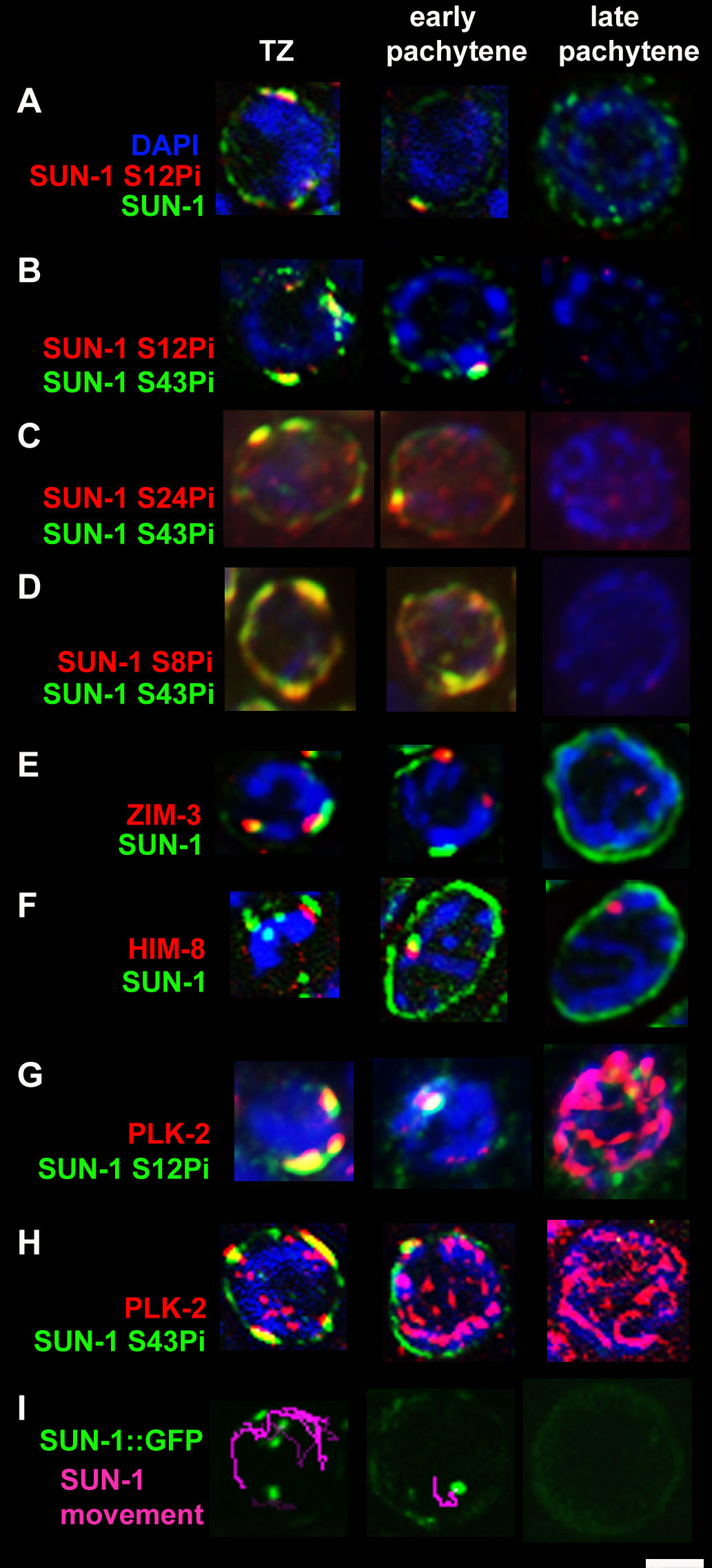

Supplement: Figure S1 — Colocalization of SUN-1 aggregates, SUN-1 phosphorylation signals, ZIMs, and PLK-2, as observed in this and previous studies [11], [12], [14], [30]. Representative wild-type hermaphrodite nuclei from TZ (left), early pachytene (middle), and late pachytene (right). All nuclei were stained with DAPI (blue). (A) SUN-1 (green) forms aggregates in the TZ; these aggregates are phosphorylated on SUN-1 S12 (red). SUN-1 not within aggregates is not phosphorylated on S12. In early pachytene, only one aggregate remains, and it is phosphorylated on S12. In late pachytene, aggregates and SUN-1 phosphorylation on S12 are gone. (B) Phosphorylation of SUN-1 S12 (red) and S43 (green) are seen at the same time. S43 phosphorylation pattern highlights the entire nuclear envelope, including the SUN-1 aggregates at chromosome ends. In early pachytene, SUN-1 S43 remains phosphorylated on the last aggregate (where it overlaps with S12 phosphorylation) and on the entire nuclear envelope. In late pachytene, S43 and S12 phosphorylation is gone. S43 phosphorylation pattern (green) overlaps with S24 (C, red) and S8 (D, red). (E) Autosomal pairing-center binding protein ZIM-3 (red) always colocalizes with SUN-1 (green) aggregates in the TZ. In early pachytene, ZIM-3 does not colocalize with the last remaining prominent SUN-1 aggregate. (F) HIM-8 (red), in contrast, remains colocalized with SUN-1 (green) aggregates in early pachytene. (G) PLK-2 (red) shares the localization pattern with its target phosphorylation site SUN-1 S12 (green) at SUN-1 aggregate(s) in the TZ and early pachytene. PLK-2 starts to localize to synapsing chromosomes from the TZ onwards and is found on all synapsed axes by late pachytene. (H) PLK-2 (red) and S43 phosphorylation (green) only overlap at SUN-1 aggregates. (I) Displacement tracks of SUN-1 aggregates represent 2D plotted chromosome end movements over 3 min. Scale bar, 2 µm. (TIF) [file pgen.1003335.s001.tif]

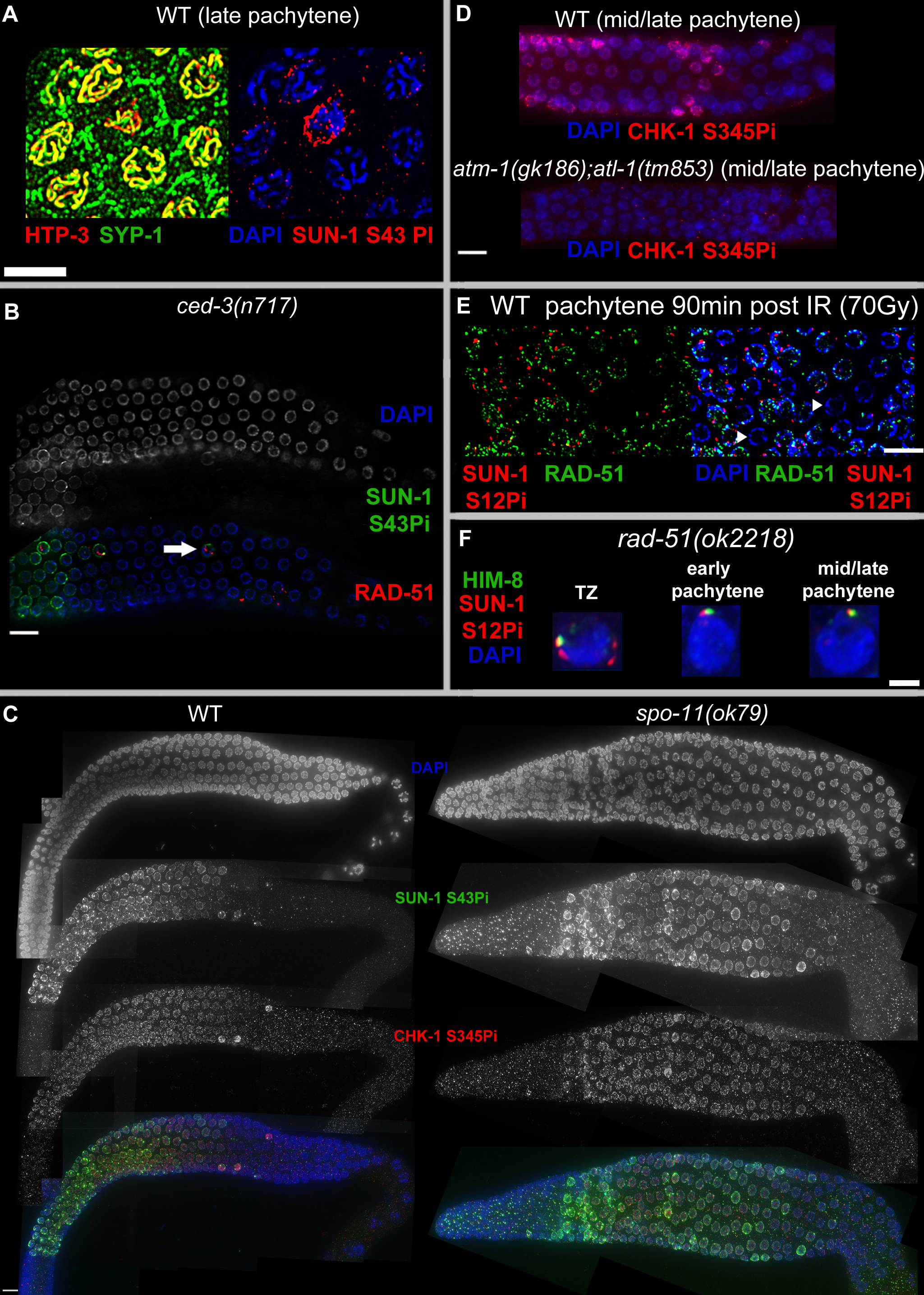

Supplement: Figure S2 — Occasional chromatin clustering in pachytene correlates with synaptic failure. (A) Mid/late pachytene nuclei of a wild-type hermaphrodite gonad stained with anti-SYP-1 (left, green), anti-HTP-3 (left, red), anti-SUN1 S43Pi (right, red), and DAPI (right, blue). Nucleus in the middle with SUN1 phosphorylation has clustered chromatin and partly unsynapsed chromatin, in contrast to the surrounding nuclei with pachytene characteristics. Scale bar, 5 µm. (B) Nuclei with high numbers of RAD-51 foci, phosphorylated SUN-1, and clustered chromatin are also present in mid/late pachytene in apoptosis-deficient mutants. ced-3(n717) mutant hermaphrodite gonad stained with DAPI (top, blue in merged), anti-SUN-1 S43Pi (middle, green in merged), and anti-RAD-51 (bottom, red in merged). Arrow indicates nucleus in late pachytene zone with clustered chromatin, phosphorylated SUN-1, and abundant RAD-51 signal. (C) SUN-1 phosphorylation correlates with phosphorylated CHK-1 in wild type and mutants. WT (left) and spo-11(ok79) mutant (right) hermaphrodite gonad stained with DAPI (top, blue in merged), anti-SUN-1 S43Pi (middle, green in merged), and anti-CHK-1 S345Pi (bottom, red in merged). (D) CHK-1 phosphorylation depends on ATM/ATL. WT (top) and atm-1(gk186); atl-1(tm853) mid/late pachytene section; DAPI (blue) and antiCHK-1 S345Pi (red). (E) Irradiation-induced damage correlates with persistent SUN-1 phosphorylation. Pachytene WT hermaphrodite gonad dissected 27 h after 70 Gy gamma irradiation; anti-SUN-1 S12Pi (red), anti-RAD-51 (green), and DAPI (blue, right). Cells devoid of RAD-51 signal are also devoid of SUN-1 S12Pi signal (arrowheads). (F) Representative nuclei from TZ (left) early pachytene (middle) and mid/late pachytene (right) in rad-51(ok2218). SUN-1 aggregates phosphorylated on S12 (red) colocalize with the HIM-8 signals (green) in all three zones. Scale bars: 5 µm in (A),10 µm in (B–E) and 2 µm in (F). (TIF) [file pgen.1003335.s002.tif]

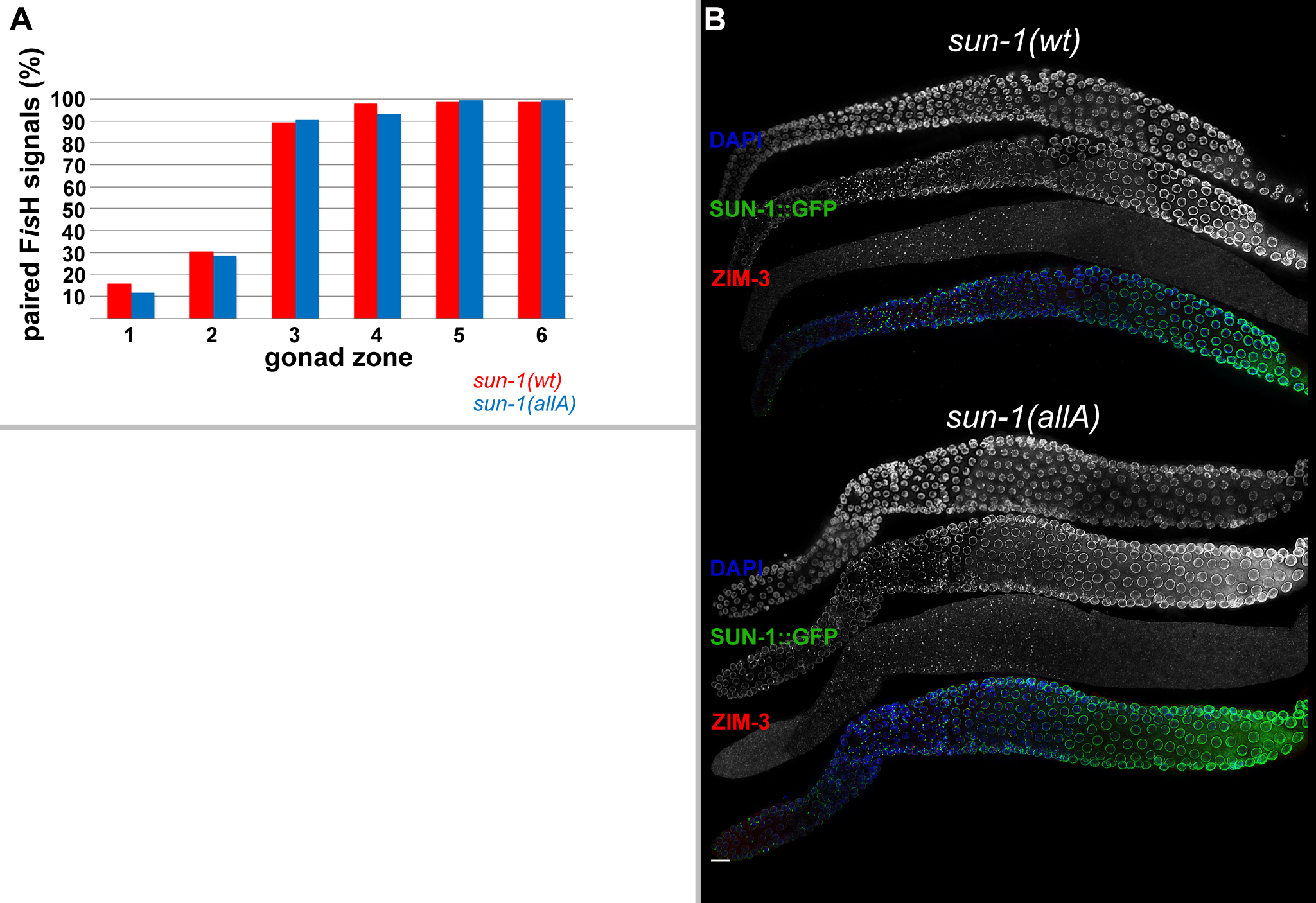

Supplement: Figure S3 — Homologous pairing and ZIM loading in a SUN-1 nonphosphorylatable mutant follow wild-type kinetics. (A) Pairing of chromosome V in sun-1(allA); sun-1(ok1282) mutant and sun-1(wt); sun-1(ok1282) was evaluated by FISH, highlighting 5S rDNA. Percentages of paired signals were assessed after dividing gonads into six zones of equal length. (B) sun-1(wt); sun-1(ok1282) and sun-1(allA); sun-1(ok1282) (bottom) hermaphrodite gonads stained with DAPI (top, blue in merged), anti-GFP (middle, green in merged), and anti-ZIM-3 (bottom, red in merged). Scale bar, 10 µm. (TIF) [file pgen.1003335.s003.tif]

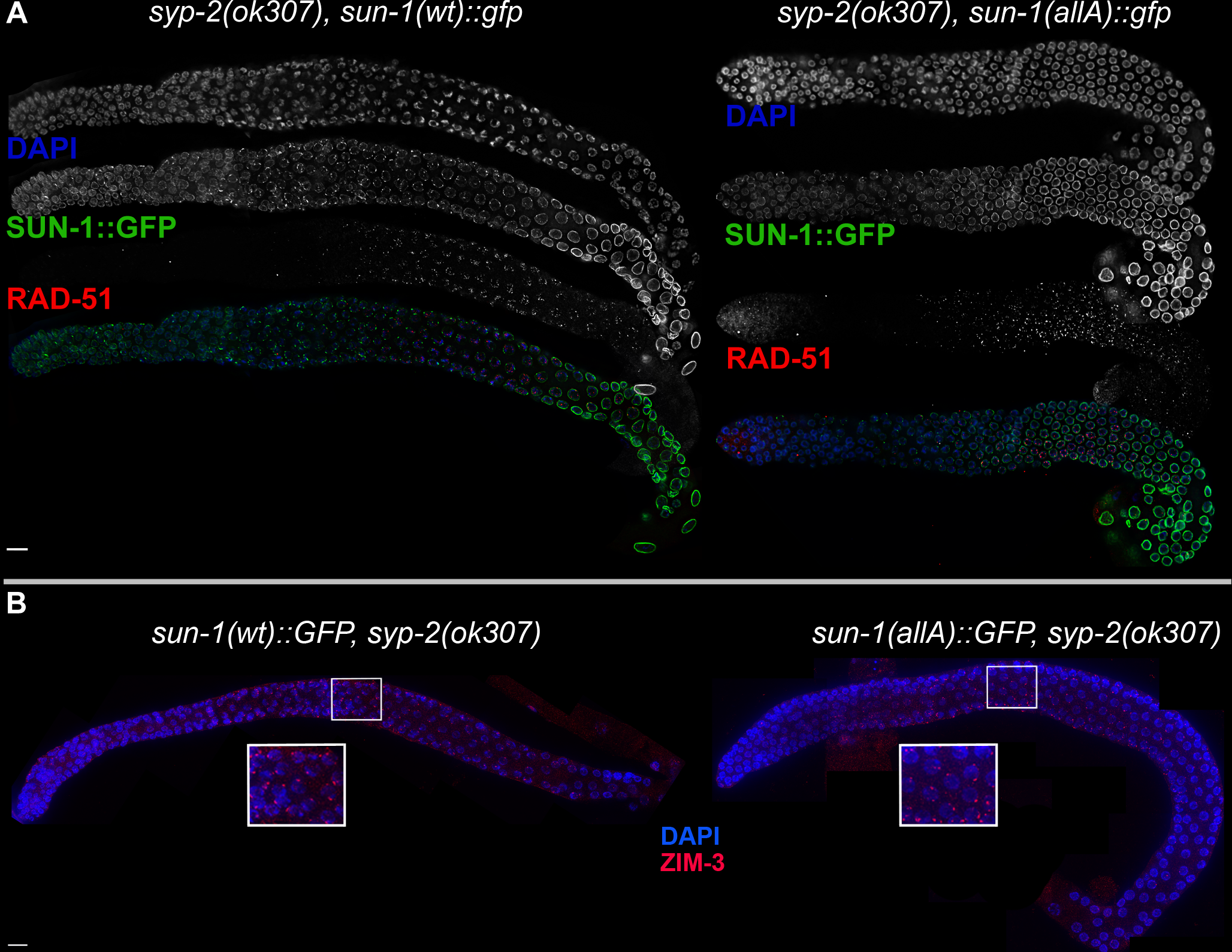

Supplement: Figure S4 — Nonphosphorylatable sun-1 mutations do not affect DSB repair kinetics in a syp-2 mutant. (A) sun-1(wt); sun-1(ok1282); syp-2(ok307) (left) and sun-1(allA); sun-1(ok1282); syp-2(ok307) (right) hermaphrodite gonads stained with DAPI (top, blue in merge), anti-GFP (middle, green in merge), and anti-RAD-51 (bottom, red in merge). (B) SUN-1 phosphorylation is dispensable for stable loading of ZIMs. sun-1(wt); sun-1(ok1282); syp-2(ok307) (left) and sun-1(allA); sun-1(ok1282); syp-2(ok307) (right) hermaphrodite gonads stained with DAPI (blue) and anti-ZIM-3 (red). Scale bars, 10 µm. (TIF) [file pgen.1003335.s004.tif]

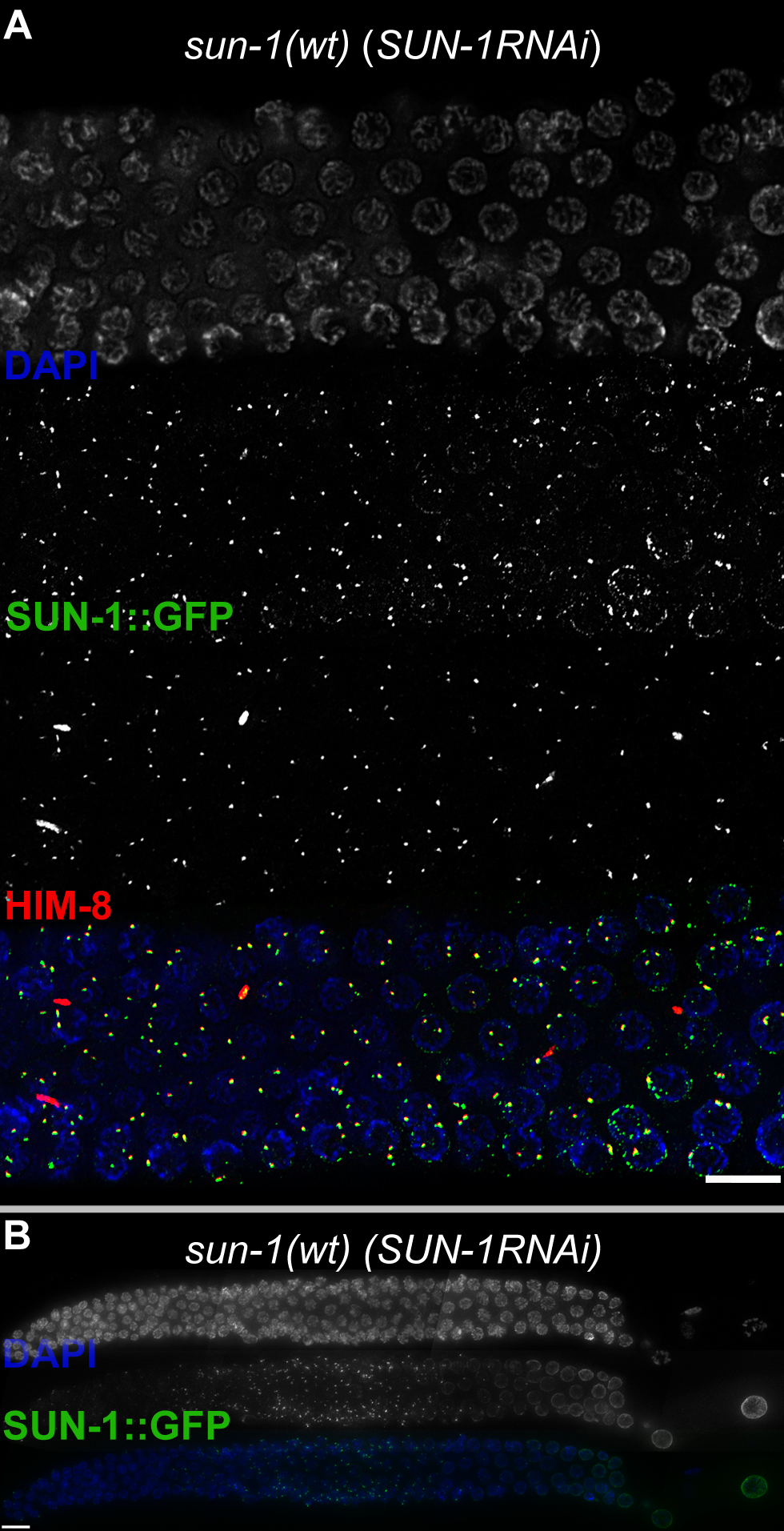

Supplement: Figure S5 — Decreased SUN-1 protein leads to failure of chromosome pairing. (A) Pachytene hermaphrodite sun-1(wt); sun-1(ok1282) gonads subjected to sun-1 RNAi stained with DAPI (top, blue in merge), anti-GFP (middle, green in merge), and anti-HIM-8 (bottom, red in merge). (B) Reduction in SUN-1 protein dosage leads to prolonged SUN-1 aggregation and chromatin clustering. sun-1(wt); sun-1(ok1282) hermaphrodite gonads subjected to sun-1 RNAi stained with DAPI (top, blue in merge) and anti-GFP (bottom, green in merge). Scale bar, 10 µm. (TIF) [file pgen.1003335.s005.tif]
