# Supplementary material for: Matefin/SUN-1 Phosphorylation Is Part of a Surveillance Mechanism to Coordinate Chromosome Synapsis and Recombination with Meiotic Progression and Chromosome Movement
Source: PLoS Genet. 2013 Mar 7;9(3):e1003335. doi: 10.1371/journal.pgen.1003335 (PMC3591285; doi:10.1371/journal.pgen.1003335)
Supplement: Table S1 — SUN-1 aggregate behavior in TZ nuclei of sun-1(allA). SUN-1::GFP aggregates recorded by in vivo time-lapse microscopy in TZ nuclei of sun-1(wt) and sun-1(allA) followed by 2D plotting for manual analysis. Duration, 3 min. Variations correspond to the standard deviations. Two-tailed t-test for fusion events, 0.107 and for splitting events, 0.103. n, represents number of independently tracked one minute sequences. (DOCX) [file pgen.1003335.s006.docx]

**Table S1.**

|  | Average number of aggregates | *n* | Fusion events/min/nucleus | Splitting events/min/nucleus | *n* |
| --- | --- | --- | --- | --- | --- |
| *sun-1(wt)* | 4.8 ± 0.7 | 252 | 1.9 ± 1.2 | 2 ± 1.4 | 20 |
| *sun-1(allA)* | 5.2 ± 1.3 | 180 | 2.7 ± 1.6 | 2.8 ± 1.7 | 14 |
