# Supplementary material for: Matefin/SUN-1 Phosphorylation Is Part of a Surveillance Mechanism to Coordinate Chromosome Synapsis and Recombination with Meiotic Progression and Chromosome Movement
Source: PLoS Genet. 2013 Mar 7;9(3):e1003335. doi: 10.1371/journal.pgen.1003335 (PMC3591285; doi:10.1371/journal.pgen.1003335)
Supplement: Table S2 — Relative duration of leptotene/zygotene, early pachytene, and middle/late pachytene in sun-1 phosphosite mutants during oogenesis. Relative duration of meiotic stages were assessed by quantifying cell rows in the meiotic part of the gonad according to the following criteria: more than one SUN-1 aggregate (“TZ”), one or more SUN-1 aggregates (“aggregate zone”, TZ plus early pachytene), or no aggregates (“zone without aggregates”). When >50% of nuclei in a cell row met one of the three criteria, the cell row was scored as such. Percentages ± standard deviation shown represent numbers normalized to gonad length from meiotic entry to beginning of cellularization (diplotene stage in the wild type). *p<0.01 between the reference lines sun-1(wt) and syp-2; sun-1(wt) and the respective mutant lines in two-tailed t-test. n, number of gonads counted. (DOCX) [file pgen.1003335.s007.docx]

**Table S2.**

|  | | TZ (%) | Aggregate zone (%) (TZ + early pachytene) | Zone w/o aggregates (%) (mid/late pachytene) | *n* |
| --- | --- | --- | --- | --- | --- |
| *sun-1(wt)* | 21.4 ± 5.0 | | 51.1 ± 6.4 | 48.9 ± 6.4 | 8 |
| *sun-1(S12E)* | 31.8 ± 4.0* | | 47.1 ± 4.1 | 52.9 ± 4.1 | 10 |
| *sun-1(6E)* | 33.9 ± 10.6* | | 58.0 ± 4.5 | 42.0 ± 4.5 | 8 |
| *sun-1(S12A)* | 22.0 ± 3.6 | | 50.9 ± 6.1 | 49.1 ± 6.1 | 10 |
| *sun-1(allA)* | 34.2 ± 5.5* | | 50.1 ± 4.3 | 49.9 ± 4.3 | 8 |
| *syp-2; sun-1(wt)* | 79.1 ± 2.1 | | 81.9 ± 3.8 | 18.1 ± 3.8 | 8 |
| *syp-2; sun-1(S12E)* | 82.3 ± 6.4 | | 83.1 ± 4.8 | 16.9 ± 4.8 | 8 |
| *syp2; sun-1(6E)* | 77.7 ± 10.8 | | 82.0 ± 2.9 | 18.0 ± 2.9 | 8 |
| *syp2; sun1(S12A)* | 76.0 ± 6.0 | | 79.6 ± 6.1 | 20.4 ± 6.1 | 10 |
| *syp-2; sun-1(allA)* | 48.7 ± 5.3* | | 58.7 ± 5.9* | 41.3 ± 5.9* | 8 |
