# Supplementary material for: Matefin/SUN-1 Phosphorylation Is Part of a Surveillance Mechanism to Coordinate Chromosome Synapsis and Recombination with Meiotic Progression and Chromosome Movement
Source: PLoS Genet. 2013 Mar 7;9(3):e1003335. doi: 10.1371/journal.pgen.1003335 (PMC3591285; doi:10.1371/journal.pgen.1003335)
Supplement: Table S3 — Number of viable offspring and hatch rate of nonphosphorylatable sun-1 mutant. L4 hermaphrodites were irradiated (90 Gy) or not irradiated, and the viability of eggs between 2 and 48 h after irradiation was assessed at 20°C. *p<0.001 in a two-tailed t-test between sun-1(wt) and sun-1(allA). n, number of animals evaluated. (DOCX) [file pgen.1003335.s008.docx]

**Table S3.**

|  | Number of viable offspring/hermaphrodites | Hatch rate (%) | *n* |
| --- | --- | --- | --- |
| *sun-1(wt)* non-IR 2–24 h | 29.4 ± 9.8 | 99.7 ± 0.8 | 20 |
| *sun-1(wt)* 90 Gy IR 2–24 h | 15.3 ± 4.9 | 65.2 ± 11.7 | 20 |
| *sun-1(allA)* non-IR 2–24 h | 19.1 ± 10.8 | 98.1 ± 2.9 | 20 |
| *sun-1(allA)* 90 Gy IR 2–24 h | 8.8 ± 10.1 | 55.36 ± 29.7 | 20 |
| *sun-1(wt)* non-IR 24–48 h | 73.3 ± 17.1 | 99.9 ± 0.5 | 20 |
| *sun-1(wt)* 90 Gy IR 24–48 h | 34.7 ± 10.4* | 70.7 ± 11.6* | 20 |
| *sun-1(allA)* non-IR 24–48 h | 82.5 ± 11.5 | 98.6 ± 1.5 | 20 |
| *sun-1(allA)* 90 Gy IR 24–48 h | 15.0 ± 11.2* | 46.8 ± 25.3* | 20 |
