# Supplementary material for: Matefin/SUN-1 Phosphorylation Is Part of a Surveillance Mechanism to Coordinate Chromosome Synapsis and Recombination with Meiotic Progression and Chromosome Movement
Source: PLoS Genet. 2013 Mar 7;9(3):e1003335. doi: 10.1371/journal.pgen.1003335 (PMC3591285; doi:10.1371/journal.pgen.1003335)
Supplement: Table S4 — Number of apoptotic corpses per gonad arm. Age-matched hermaphrodites were stained with SYTO 12. Positive nuclei were scored as apoptotic corpses. Variations indicate the standard error of the mean (SEM). Each genotype was analyzed in at least three independent experiments. n, number of scored gonad arms. (DOCX) [file pgen.1003335.s009.docx]

**Table S4.**

|  | Apoptotic corpses/gonad arm | *n* |
| --- | --- | --- |
| WT | 3.5 ± 0.17 | 56 |
| *sun-1(wt)* | 3.37 ± 0.28 | 40 |
| *sun-1(6E)* | 3.35 ± 0.016 | 136 |
| *sun-1(allA)* | 3.56 ± 0.16 | 123 |
| *syp-2; sun-1(wt)* | 14.72 ± 0.72 | 67 |
| *syp-2; sun-1(allA)* | 13.24 ± 0.58 | 84 |
| *syp-2; sun-1(6E)* | 10.25 ± 0.48 | 61 |
