# Supplementary material for: Matefin/SUN-1 Phosphorylation Is Part of a Surveillance Mechanism to Coordinate Chromosome Synapsis and Recombination with Meiotic Progression and Chromosome Movement
Source: PLoS Genet. 2013 Mar 7;9(3):e1003335. doi: 10.1371/journal.pgen.1003335 (PMC3591285; doi:10.1371/journal.pgen.1003335)
Supplement: Table S5 — Relative duration of leptotene/zygotene (TZ) and pachytene in sun-1 phosphosite mutants during male meiosis. Relative durations of meiotic stages were assessed by quantifying cell rows in the meiotic part of the gonad according to the following criteria: more than one SUN-1 aggregate (“TZ”) or no aggregates (“pachytene”). When >50% of nuclei in a cell row met these criteria, the cell row was scored as such. Percentages ± standard deviation shown represent numbers normalized to gonad length from meiotic entry to beginning of diplotene. n, number of gonads counted. (DOCX) [file pgen.1003335.s010.docx]

**Table S5.**

|  | TZ (%) | Pachytene (%) | *n* |
| --- | --- | --- | --- |
| *sun-1(wt)* | 41.8 ± 7.0 | 48.2 ± 7.0 | 10 |
| *sun-1(S12E)* | 52.3 ± 8.6 | 47.7 ± 8.6 | 12 |
| *sun-1(6E)* | 52.9 ± 8.1 | 47.1 ± 8.1 | 10 |
| *sun-1(S12A)* | 39.5.3 ± 3.1 | 60.5 ± 3.1 | 10 |
| *sun-1(allA)* | 39.7 ± 5.7 | 60.3 ± 5.7 | 10 |
